# Three Novel Spider Genomes Unveil Spidroin Diversification and Hox Cluster Architecture: *Ryuthela nishihirai* (Liphistiidae), *Uloborus plumipes* (Uloboridae) and *Cheiracanthium punctorium* (Cheiracanthiidae)

**DOI:** 10.1111/1755-0998.14038

**Published:** 2024-10-22

**Authors:** Yannis Schöneberg, Tracy Lynn Audisio, Alexander Ben Hamadou, Martin Forman, Jiří Král, Tereza Kořínková, Eva Líznarová, Christoph Mayer, Lenka Prokopcová, Henrik Krehenwinkel, Stefan Prost, Susan Kennedy

**Affiliations:** ^1^ Department of Biogeography Trier University Trier Germany; ^2^ Evolutionary Genomics Unit Okinawa Institute of Science and Technology Okinawa Japan; ^3^ LOEWE‐Centre for Translational Biodiversity Genomics (LOEWE‐TBG) Frankfurt am Main Germany; ^4^ Senckenberg Forschungsinstitut und Naturmuseum Frankfurt am Main Germany; ^5^ Laboratory of Arachnid Cytogenetics, Department of Genetics and Microbiology, Faculty of Sciences Charles University Prague 2 Czech Republic; ^6^ Department of Botany and Zoology, Faculty of Science Masaryk University Brno Czech Republic; ^7^ Centre for Molecular Biodiversity Research Leibniz Institute for the Analysis of Biodiversity Change Bonn Germany; ^8^ Ecology and Genetics Research Unit University of Oulu Oulu Finland; ^9^ South African National Biodiversity Institute National Zoological Garden Pretoria South Africa; ^10^ Natural History Museum Vienna Central Research Laboratories Vienna Austria

**Keywords:** assembly, chromosome, Hi‐C, karyotype, Mesothelae, spider silk

## Abstract

Spiders are a hyperdiverse taxon and among the most abundant predators in nearly all terrestrial habitats. Their success is often attributed to key developments in their evolution such as silk and venom production and major apomorphies such as a whole‐genome duplication. Resolving deep relationships within the spider tree of life has been historically challenging, making it difficult to measure the relative importance of these novelties for spider evolution. Whole‐genome data offer an essential resource in these efforts, but also for functional genomic studies. Here, we present de novo assemblies for three spider species: *Ryuthela nishihirai* (Liphistiidae), a representative of the ancient Mesothelae, the suborder that is sister to all other extant spiders; *Uloborus plumipes* (Uloboridae), a cribellate orbweaver whose phylogenetic placement is especially challenging; and *Cheiracanthium punctorium* (Cheiracanthiidae), which represents only the second family to be sequenced in the hyperdiverse Dionycha clade. These genomes fill critical gaps in the spider tree of life. Using these novel genomes along with 25 previously published ones, we examine the evolutionary history of spidroin gene and structural hox cluster diversity. Our assemblies provide critical genomic resources to facilitate deeper investigations into spider evolution. The near chromosome‐level genome of the ‘living fossil’ *R. nishihirai* represents an especially important step forward, offering new insights into the origins of spider traits.

## 1 Introduction

**FIGURE 1:**
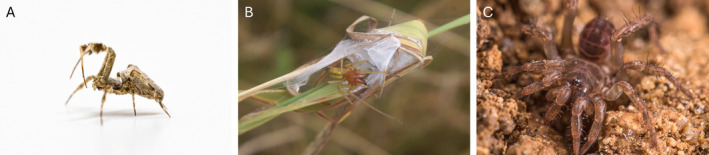
Photographs of all three sequenced species. (A) *Uloborus plumipes*; (B) Male *Cheiracanthium punctorium* defending its shelter sac; (C) *Ryuthela nishihirai*. Copyright from left to right: Yannis Schöneberg, Till Macher, Ales Bucek.

Spiders are a hyperdiverse taxon with approx. 52,000 recognised species in 134 families and roughly 900 newly described species every year (Gloor et al. 2024). They have colonised terrestrial habitats on all continents except Antarctica and are one of the most common and abundant predators worldwide (Foelix 2011). They play an important role in trophic networks and are estimated to consume 400–800 million tons of insect biomass every year, though some are even able to feed on small vertebrates (Foelix 2011; Nyffeler and Birkhofer 2017). Although spiders are one of the most successful animal taxa, whole‐genome data are limited to a handful of families, most of these in the superfamily Araneoidea. To provide a broader scale perspective on genome evolution in spiders, we *de novo* assembled three genomes, which were selected to cover previously unrepresented lineages. Our assembly of *Uloborus plumipes* represents the first for this species and second for the family Uloboridae (Figure 1A). The family's members lost their venom apparatus but gained a unique way of expressing venom in their midguts (Peng et al. 2023). The assembly for the yellow sac spider *Cheiracanthium punctorium* is the first for the family Cheiracanthiidae (Figure 1B). Next to salticids, this is only the second family sequenced in the Dionycha clade. This clade comprises well over 25% of all spider species described today. Hence, the assembly offers an important resource to study the diversity and phylogeny of this extraordinarily species‐rich clade. The third species we sequenced, *Ryuthela nishihirai* (Liphistiidae), is the first reference genome of Mesothelae, the suborder that diverged over 350 Mya and forms the sister clade to all other living spiders (Kulkarni, Wood, and Hormiga 2023, Figure 1C). Species of this suborder are often referred to as ‘living fossils’ because they retained several ancestral characters that were lost or modified in all other recent spiders, for example, two pairs of book lungs, 7–8 undifferentiated spinnerets and a segmented abdomen (Haupt 2003). Their unique life history traits and phylogenetic position make this genome assembly a key resource to understand the evolution and patterns of diversity in spiders. These new genome assemblies fill major gaps in the spider genome phylogeny. We thus use them in concert with 25 publicly available spider assemblies to perform an initial analysis of two potential key developments in spider evolution: spidroin and hox cluster diversification.

The evolutionary success of spiders has been attributed to several evolutionary novelties, for example, their venom, their use of tracheae as respiratory organ, as well as their well‐developed and unique sensory organs (Barth 1997; Dimitrov and Hormiga 2021 and references herein). Yet, another unique trait frequently attributed to the evolutionary success of spiders is their capacity to employ silk for a broad range of applications, for example, web building, protecting their offspring and even passive aerial dispersal (ballooning) (Craig 2003; Foelix 2011). Spider silk possesses extraordinary mechanical properties and is extensively studied for its potential in biomedical and biomechanical applications (Bakhshandeh et al. 2021; Arakawa et al. 2022). While considerable research has been devoted to the morphology, mechanics, physiology and biochemistry of these traits, their genetic basis is still not well‐understood. Considering this background, genomic resources for spiders are much needed. Due to the large and often complex genomes of spiders, these resources have long been hard to assemble, leaving many critical gaps of available reference genomes across the spider tree of life. Spider silk, indisputably one of the most important evolutionary novelties of the group, has been particularly difficult to study genetically, due to the large size and repetitive nature of silk genes. Its main constituents are the so‐called spidroin proteins, which consist of a N‐terminal domain (NTD) and a C‐terminal domain (CTD), with a repetitive region in between. The terminal domains play a vital role in assembling the silk polymer, while the large repetitive region determines the mechanical properties (Blamires et al. 2017). This repetitive region has made it difficult to sequence full‐length spidroins, and only modern long‐read sequencing technologies have enabled the large‐scale study of spidroins in their full length. Therefore, it is not surprising that most research to date has focussed on the terminal regions or single species (e.g., Babb et al. 2017; Arakawa et al. 2022; Fan et al. 2023). To date, no study has examined full‐length spidroin diversity using a data set spanning the whole spider tree of life. Such a data set of full‐length spidroins might provide insights into the evolution of spider silk properties and could reveal a detailed picture of its role in spider diversification.

Recently, a potential whole‐genome duplication (WGD) in the ancestor of Arachnopulmonates (i.e., the clade formed by scorpions, spiders, whip scorpions and whip spiders) has been discussed as a key event in arachnid evolution (Schwager et al. 2017; Sharma 2023). Arachnopulmonates possess two copies of the homeobox (hox) cluster (Schwager et al. 2017; Leite et al. 2018; Harper et al. 2021). The genes from these clusters have numerous regulatory functions in development and determine the body plan of many bilaterians (e.g., Holland, Booth, and Bruford 2007; Larroux et al. 2007; Holland et al. 2017). Complex regulatory mechanisms controlling the expression of adjacent genes make the structure of the cluster highly conserved, and seemingly, small mutations can lead to drastic changes in the body plan (Averof and Patel 1997; Casares and Mann 1998; Abzhanov, Popadic, and Kaufman 1999). Therefore, the duplication of the hox cluster in Arachnopulmonates constitutes a significant evolutionary process. Such gene duplications are often associated with sub‐ and neofunctionalisation, and considered drivers of diversification (Ohno 1970; Lynch and Conery 2000). Indeed, Leite et al. (2018) found evidence for considerable neofunctionalisation in the spider hox clusters, and recently, Aase‐Remedios et al. (2023) showed considerable structural plasticity of the second hox cluster analysing araneomorph spiders. This raises the question of whether the hox clusters already show such structural variation in early branching spiders, and what role they played in spider diversification.

In this study, we present three *de novo* assembled genomes as key genomic resources to understand the evolution and diversity of spiders. We use the novel and 25 publicly available spider genome assemblies for an initial analysis of two gene families that may have played a critical role in spider evolution: spidroin and hox genes.

## 2 Methods

### 2.1 Karyotyping and Genome Size Estimation

When assembling genomes, it is helpful to know their approximate size, repeat content, heterozygosity and chromosome number. This helps to determine the right settings for the assembler and provides estimates for quality control of the final assembly. Therefore, we karyotyped all three species and estimated the genome sizes and other genome characteristics using two different methods: Flow Cytometry and K‐mer Analysis.

To obtain chromosome plates, gonads of a subadult male of *C. punctorium* (Hrádek, Srch, Czech Republic), two male juveniles of *R. nishihirai* (one from Sueyoshi, another one from Ryutan, Okinawa Island, Ryukyu islands, Japan) and a female juvenile of *U. plumipes* (garden centre, Ravensburg, Germany) were used. We dissected the juvenile specimens and used the presence of ovaries or testes to determine their sex. Chromosomes were prepared and Giemsa‐stained according to the method described in Dolejš et al. (2011). Tissues were hypotonised with 0.075M KCl for 20 min (*Cheiracanthium* and *Uloborus*) or 30 min (*Ryuthela*), and fixed three times (6, 10, and 20 min) using ethanol: acetic acid fixation (3:1). The slides were stained with 5% Giemsa solution in modified Sörensen buffer (4.54 g KH_2_PO_4_, 4.75 g Na_2_HPO_4_ 12H_2_O, 1000 mL H_2_O, pH 6.8) for 28 min. Mitotic metaphases were used for chromosome measurements using the ImageJ software (https://imagej.net/ij/). Relative chromosome length was estimated as a percentage of the total chromosome length of the haploid set (including sex chromosomes). For *Ryuthela*, the chromosomal plates only allowed to determine the diploid number of chromosomes.

To estimate the genome size and proportion of GC bases, we used flow cytometry on a subadult male of *C. punctorium* (Výhon, Židlochovice, Czech Republic) and an adult female of *U. plumipes* (garden centre, Ravensburg, Germany). We did not conduct flow cytometry for *R. nishihirai* due to the limited number of specimens. We used the method described in Král et al. (2019), which includes two fluorochromes. Intercalating nonspecific propidium iodide was used to calculate the genome sizes. Propidium iodide and an AT‐specific 4′,6‐diamidino‐2‐phenylindole (DAPI) were used for calculating the AT:GC ratio. We used the following standards: man's leucocytes (2C = 6.055 Gbp, GC = 43.60%, Doležel, Sgorbati, and Lucretti 1992) for *U. plumipes* and a fresh leaf of *Bellis perennis* (2C = 3.090 Gbp, GC = 39.54%, Veselý et al. 2012) for *C. punctorium*, respectively.

To estimate the genome size, repeat content, heterozygosity and GC‐content *in silico*, we performed a K‐mer analysis for all three specimens using short read data generated for whole‐genome sequencing (see ‘DNA Extraction & Sequencing’ below). We first ran Jellyfish v2.2.10 with a K‐mer length of 21 bp (Marcais and Kingsford 2011), and subsequently GenomeScope v2.0 (Ranallo‐Benavidez, Jaron, and Schatz 2020). Results can be found in Table S1.

### 2.2 DNA Extraction and Sequencing

Specimens of *C. punctorium* (for DNA sequencing) were collected on campus of Trier University, Trier, Germany, on 10 October 2021, and immediately stored over liquid nitrogen (−160°C). We collected six female individuals of *U. plumipes* in the greenhouses of a garden centre located in Mainaschaff, Germany, on 24 November 2022. The specimens were initially stored at −80°C and later transferred to liquid nitrogen storage (−160°C). The individuals for *R. nishihirai* were collected in a cave near Yomitan on Okinawa, Japan, in June, snap‐frozen and stored at −80°C, and later transferred to liquid nitrogen storage (−160°C). We determined the species of the specimens morphologically and checked CO1 sequences after sequencing. After DNA‐extraction (see below), we deposited the specimens in the LIB‐Biobank at the Research Museum Alexander König, Bonn, Germany, under the following IDs: ZFMK‐TIS‐93302—ZFMK‐TIS‐93305. Due to their small size and destructive dissection, the specimens for *U. plumipes* could not be deposited.

For DNA extraction, we tried to minimise contamination with bacteria living in the digestive tract using only the prosoma and legs for each species, as a large part of spider's digestive system is situated in the opisthosoma. For *R. nishihirai* and *C. punctorium*, we used the Monarch HMW DNA Extraction Kit for Tissue v2.1_4/21 (New England Biolabs NE). As we could not achieve satisfying results using this kit for *U. plumipes*, we slightly modified the protocol of the Puregene Tissue Kit (Qiagen) to yield high‐molecular‐weight DNA. Vortexing was replaced with 25 slow, gentle inversions to ensure mixing without damaging DNA. In addition, GlycoBlue coprecipitant (Thermofisher) was used to maximise yield. DNA integrity and purity were evaluated using a Nanodrop, Quantos Fluorometer and an Agilent Tapestation 2200 (Agilent technologies) using Genomic DNA ScreenTapes. After DNA extraction, we prepared continuous long‐read (CLR) sequencing libraries for *U. plumipes* and *C. punctorium* using the SMRTbell Express Template Prep Kit 2.0 (Pacific Biosciences, Menlo Park, CA, USA). The sequencing libraries were evaluated using the Agilent Tapestation 2200 and the Quantos Fluorometer. For both species, CLR data were generated on a PacBio Sequel II at the Radboud University Medical Center, Nijmegen, Netherlands. Since CLR data show higher read error rates than Illumina or DNBSEQ short‐read sequencing, we performed a short‐read polishing step. To do so, the genomes were additionally sequenced with highly accurate short‐reads with 50X coverage. We used the same DNA extractions and individuals as for the CLR sequencing described above and sent the samples to BGI Tech Solutions (Europe), Rotterdam, Netherlands, for DNBSEQ Normal DNA library preparation and paired‐end DNBSEQ sequencing on a DNBSEQ‐T7 machine (MGI Tech Co., Shenzhen, China). The DNA extraction for *R. nishihirai* was sent to the Uppsala Genome Center, Uppsala, Sweden for library preparation and circular consensus sequencing (CCS) on a PacBio Sequel II.

To be able to assemble the genomes to chromosome level, we prepared Arima Hi‐C libraries for all three species. We used the Arima Genomics High Coverage Hi‐C Kit following the User Guide for Animal Tissues and the Arima Hi‐C+ Kit User Guide for Library Preparation with the Arima Library Prep Module for the *Ryuthela*. For the remaining two species, we used the Accel‐NGS 2S PLUS DNA Library Kit. As specimens for all species were rather small, we had to use different specimens for Hi‐C than for shotgun sequencing. However, we selected specimens from the same localities. Similar to the PacBio sequencing, we only used the legs of the specimens for Hi‐C library preparation to avoid bacterial contamination from the digestive system. Library QC was performed using the Quantos Fluorometer and the Agilent Tapestation 2200 as described in the Arima user guide. The libraries were sent to Novogene Co. Ltd. (Beijing, China) for sequencing with 60× target read coverage on an Illumina NovaSeq 6000.

### 2.3 Genome Assemblies

For the assemblies based on CLR data *U. plumipes* and *C. punctorium*. As spiders seem to have complex genomes, we tried various parameter settings (Tables S2 and S3) and evaluated the results using Quast v5.2.0 and BUSCO v5.4.5 on the arachnida_odb10 data set (Gurevich et al. 2013; Seppey, Manni, and Zdobnov 2019). The best assemblies were achieved with the following settings: *U. plumipes* (wtdbg2 ‐AS 2 ‐K 2000 ‐e 2 ‐p 16 ‐k 1 ‐align‐dovetail 3072); *C. punctorium* (wtdbg2 ‐p 16 ‐k 1 ‐K 2000 ‐AS 2 ‐e 3 ‐R ‐t 72 ‐L 3000 ‐aln‐dovetail 2048) (Ruan and Li 2020). Subsequently, we ran three rounds of Flye v2.9.1 long‐read polishing (Lin et al. 2016). To avoid overpolishing, we chose the best round based on the statistics generated using Quast v5.2.0 and BUSCO v5.4.5 on the arachnida_odb10 BUSCO gene set (Gurevich et al. 2013; Seppey, Manni, and Zdobnov 2019). To remove sequencing errors introduced by the error‐prone CLR data, we used accurate short‐reads for polishing. We performed three rounds of short‐read polishing using the wtdbg2‐racon‐pilon‐script (available on https://github.com/schellt/wtdbg2‐racon‐pilon/blob/master/wtdbg2‐racon‐pilon.pl) by only executing the pilon steps. The script used bwa mem v0.7.17 and Samtools v1.16.1 for mapping the reads against the assembly and Pilon v1.24‐0 to perform the actual short read polishing (Li and Durbin 2009; Walker et al. 2014; Danecek et al. 2021). Each round was assessed using Quast v5.2.0 (Gurevich et al. 2013) and BUSCO v5.4.5 (Seppey, Manni, and Zdobnov 2019) on the arachnida_odb10 BUSCO genes, and the best round was chosen for downstream analyses. Since we had CCS‐Data for the *Ryuthela*, we chose a different assembly strategy. We utilised Hifiasm v0.19.5 with standard settings (Cheng et al. 2021) using the combined data from all seven SMRT‐cells of HiFi‐sequencing, and Hi‐C‐sequencing. The completeness of the resulting assembly was assessed using Quast v5.2.0 (Gurevich et al. 2013), BUSCO v5.4.5 (Seppey, Manni, and Zdobnov 2019) on the arachnida_odb10 BUSCO genes.

We assessed the amount of contamination using Blobtoolkit v4.1.5 and checked for general assembly errors as well as completeness using Merqury v1.3 (Challis et al. 2020; Rhie et al. 2020). To use Blobtoolkit, we had to search all contigs against the NCBI nucleotide database using blast v2.13.0 to get a taxonomic assignment using the default settings. The coverage information was generated by mapping the long‐read data against the assembly using Minimap v2.24 (Li 2018; Danecek et al. 2021). The output data were then processed using samtools v1.17, using the subcommands *sort* and *index* to get the input files for Blobtoolkit. Then, we ran Blobtoolkit v4.1.5 and generated snail, blob and cumulative plots for each assembly using blobtk v0.4.7 (available on https://github.com/blobtoolkit/blobtk). Merqury provides a reference‐free way to assess the completeness of a genome assembly and the number of pseudoduplications. It uses K‐mer multiplicity to assess whether all K‐mers present in the read set are also present in the assembly and at which coverage. We first used Merqury's best_k.sh script to estimate the best k‐mer size, then counted K‐mers using Meryl v1.3 and subsequently ran Merqury v1.3 (Rhie et al. 2020). We used this analysis to assess whether further purging duplicated haplotypes is necessary for all our contig level assemblies. Only the assembly of *U. plumipes* showed signs of duplicated sequences. We used purge_haplotigs v1.1.2 to remove duplicated sequences based on kmer‐coverage (Roach, Schmidt, and Borneman 2018). Quality statistics of the purged assembly were generated using Quast v5.2.0 (Gurevich et al. 2013), BUSCO v5.4.5 (Seppey, Manni, and Zdobnov 2019) on the arachnida_odb10 data set and Merqury v1.3 (Rhie et al. 2020) as described above.

After finishing the contig level assemblies, we used the Arima Hi‐C‐sequencing data to scaffold the contigs into larger scaffolds and potential chromosomes. As the first step, we mapped the sequencing reads against the respective genome using the Arima Mapping Pipeline A160156 v02 (available on https://github.com/ArimaGenomics/mapping_pipeline). The pipeline first used bwa mem v0.7.17 (Li and Durbin 2009) to map the forward and reverse reads separately to the assembly. Subsequently, chimeric reads were filtered using scripts included with the pipeline, and the remaining reads were paired using samtools v1.17 (Danecek et al. 2021). Next, read groups were added using the Picard v3.0.0 (Broad Institute 2019) subcommand AddOrReplaceReadGroups. The mapped data were then used with YaHS v1.2a.2 (Zhou, McCarthy, and Durbin 2023) to perform the first round of scaffolding. The resulting data were prepared for manual curation using YaHS' v1.2a.2 ‘juicer pre’ script and subsequently juicerTools pre v2.20.00 (Durand, Shamim, et al., 2016). We then used JuiceBox v1.11.08 (Durand, Robinson, et al., 2016) for manual curation. To generate a manually curated fasta file for the second round of Hi‐C‐scaffolding, we used the script ‘juicer post’ coming with YaHS' v1.2a.2. Unfortunately, it showed that the Hi‐C‐scaffolding failed for *U. plumipes* and *C. punctorium*. Therefore, we only conducted a second round of Hi‐C‐scaffolding for *Ryuthela nishihirai*. The second round was performed as described for the first one. The results of each round were quality controlled using Quast v5.2.0 (Gurevich et al. 2013), and BUSCO v5.4.5 (Seppey, Manni, and Zdobnov 2019) on the arachnidae_odb10 BUSCO genes. The final contact map was generated using the ‘juicer pre’ script bundled with YaHS' v1.2a.2, and subsequently JuicerTools v2.20.00 pre to generate the input files for JuiceBox v1.11.08, which was used for visualisation. After Hi‐C‐scaffolding, we ran TGS‐GapCloser v1.2.1 (Xu et al. 2020) for gap‐filling using the combined CCS data. Subsequently, we ran another round of Blobtoolkit v4.1.5 (Challis et al. 2020) as described before. This revealed some contaminating Prokaryota (Figure S1). Therefore, we used the Blobtools filter command to remove contigs that had a length < 1000 bp, had less than 3× coverage or were assigned to prokaryotic taxa. Finally, the assembly was quality‐controlled using the same approaches as described above.

We generated custom repeat libraries for our three novel assemblies (*R. nishihirai*, *U. plumipes* and *C. punctorium*) using RepeatModeler v2.0.4 (Flynn et al. 2019), which utilised Recon v1.08 (Bao and Eddy 2002) and RepeatScout v1.0.6 (Price, Jones, and Pevzner 2005). These repeat libraries were then combined with the RepBase (Bao, Kojima, and Kohany 2015) invertebrate data set to create the repeat library used with RepeatMasker v4.1.5 (Smit, Hubley, and Green 2013–2015) to annotate the three assemblies.

### 2.4 Genome Data Set

For investigating the diversity of hox and spidroin genes, we built a data set from publicly available genomes as well as the assemblies reported herein, covering 28 species of spiders from 13 different families. The data set spans the whole spider tree of life including quite divergent lineages, although some important lineages are still missing (e.g., Mygalomorphae). As outgroup taxa we included two scorpions and two mites. The publicly available genomes we included were as follows: *Amaurobius ferox* (Henriques 2024), *Argiope bruennichi* (Wellcome Sanger Institute), *Caerostris darwini*, *C. extrusa* (both Kono, Ohtoshi, et al. 2021), *Centruroides sculpturatus* (Schwager et al. 2017), *C. vittatus* (Yamashita, Rhoads, and Pummill 2023), *Dermacentor andersoni* (United States Department of Agriculture 2022), *Dolomedes plantarius* (Wellcome Sanger Institute 2022), *Dysdera silvatica* (Escuer et al. 2022), *Ectatosticta davidi* (Fan et al. 2023), *Hylyphantes graminicola* (Zhu et al. 2022), *Ixodes scapularis* (De et al. 2023), *Latrodectus elegans* (Wang et al. 2022), *Meta bourneti* (Henriques and Sivell 2022), *Metellina segmentata* (Henriques and Sivell 2023), *Oedothorax gibbosus* (Hendrickx et al. 2022), *Parasteatoda lunata* (Oxford 2023), *P. tepidariorum* (Schwager et al. 2017), *Pardosa pseudoannulata* (Yu et al. 2024), *Stegodyphus dumicola* (Liu et al. 2019), *S. mimosarum* (Sanggaard et al. 2014), *Tetragnatha montana* (Wellcome Sanger Institute, 2023), *T. versicolor* (Adams et al. 2023), *Trichonephila clavata*, *T. clavipes* (both Kono, Nakamura, et al. 2021) and *Uloborus diversus* (Miller, Zimin, and Gordus 2022). Accessions and family information can be found in Table 1.

**TABLE 1:** Accession information for all data resources used within this study.

Family	Species	Type	NCBI‐Accession	Other
Amaurobiidae	*Amaurobius ferox*	Assembly	GCA_951213105.1	
Araneidae	*Argiope bruennichi*	Assembly	GCA_947563725.1	
Araneidae	*Caerostris darwini*	Assembly	GCA_021605075.1	
Araneidae	*Caerostris extrusa*	Assembly	GCA_021605095.1	
Buthidae	*Centruroides sculpturatus*	Assembly	GCF_000671375.1	
Buthidae	*Centruroides vittatus*	Assembly	GCA_030686945.1	
Cheiracanthiidae	*Cheiracanthium punctorium*	Assembly	GCA_038373885.1	
Dysderidae	*Dysdera silvatica*	Assembly	GCA_006491805.2	
Eresidae	*Stegodyphus dumicola*	Assembly	GCA_010614865.2	
Eresidae	*Stegodyphus mimosarum*	Assembly	GCA_000611955.2	
Hypochilidae	*Ectatosticta davidi*	Assembly		10.57760/sciencedb.06872
Ixodidae	*Dermacentor andersoni*	Assembly	GCA_023375885.2	
Ixodidae	*Ixodes scapularis*	Assembly	GCA_016920785.2	
Linyphiidae	*Hylyphantes graminicola*	Assembly	GCA_023701765.1	
Linyphiidae	*Oedothorax gibbosus*	Assembly	GCA_019343175.1	
Liphistiidae	*Ryuthela nishihirai*	Assembly	GCA_038380435.1	
Lycosidae	*Pardosa pseudoannulata*	Assembly	GCA_032207245.1	
Nephilidae	*Trichonephila clavata*	Assembly	GCA_019973975.1	
Nephilidae	*Trichonephila clavipes*	Assembly	GCA_019973935.1	
Pisauridae	*Dolomedes plantarius*	Assembly	GCA_907164885.2	
Tetragnathidae	*Meta bourneti*	Assembly	GCA_933210815.1	
Tetragnathidae	*Metellina segmentata*	Assembly	GCA_947359465.1	
Tetragnathidae	*Tetragnatha montana*	Assembly	GCA_963680715.1	
Tetragnathidae	*Tetragnatha versicolor*	Assembly	GCA_024610705.1	
Theridiidae	*Latrodectus elegans*	Assembly	GCA_030067965.1	
Theridiidae	*Parasteatoda lunata*	Assembly	GCA_949128135.1	
Theridiidae	*Parasteatoda tepidariorum*	Assembly	GCA_000365465.3	
Uloboridae	*Uloborus diversus*	Assembly	GCA_026930045.1	
Uloboridae	*Uloborus plumipes*	Assembly	GCA_038373865.1	
Atypidae	*Atypus karschi*	Transcriptome	DRR297805	
Liphistiidae	*Heptathela kimurai*	Transcriptome	DRR296880	
Liphistiidae	*Heptathela nishihirai*	Transcriptome	DRR296558	
Liphistiidae	*Heptathela nishihirai*	Transcriptome	DRR296767	
Liphistiidae	*Heptathela yakushimaensis*	Transcriptome	DRR296636	
Liphistiidae	*Heptathela yanbaruensis*	Transcriptome	DRR297950	
Liphistiidae	*Ryuthela ishigakiensis*	Transcriptome	DRR297304	
Liphistiidae	*Ryuthela sasakii*	Transcriptome	DRR296715	
Hexathelidae	*Atrax robustus*	Transcriptome	DRR297152	
Liphistiidae	*Liphistius murphyorum*	Transcriptome	DRR297690	
Theraphosidae	*Lampropelma violaceopes*	Transcriptome	DRR296550	
Theraphosidae	*Phormictopus atrichomatus*	Transcriptome	SRR8944270	

### 2.5 Identifying Spidroins and Hox Genes

Most studies examining spidroin and hox genes applied BLAST‐based approaches to identify and classify these genes (e.g., Arakawa et al. 2022; Babb et al. 2022; Wang et al. 2022; Aase‐Remedios et al. 2023), whereas we applied profile hidden Markov models (pHMM). It was shown that approaches based on pHMM outperform sequence similarity‐based approaches (e.g., BLAST) in detecting remote homologues (Johnson, Eddy, and Portugaly 2010; Skewes‐Cox et al. 2014). This is especially important when considering the long evolutionary time frame of spider evolution.

### 2.6 Spidroins

To identify spidroin genes in our data set, we used HMMER v.3.4 (hmmer.org) as a pHMM‐based ortholog finding program. The first step therefore was building the pHMMs with already available spidroin data. As training data, we downloaded the complete Silkomes Database v1.0, which represents a comprehensive database of spidroin terminal sequences (Arakawa et al. 2022). We separated the different spidroin classes (i.e., Aciniform [AcSp], Aggregate [AgSp], Cribellar [CrSp], Cyllindrical [CySp], Flagelliform [Flag], Major Ampullate [MaSp], Minor Ampullate [MiSp], Pseudoflagelliform [Pflag], Pyriform [PySp] and unclassified spidroin) and separated NTD and CTD for each of these classes. As we found a novel CTD in the Mesothelae lineage, we also built a reference data set for this CTD using *R. nishihirai* assembly and available transcriptome data (described below). The sequences of these reference sets were aligned using MAFFT v7.520 (Nakamura et al. 2018), abnormal sequences were removed manually, and the alignments were trimmed to include only highly conserved regions. We used these alignments to train pHMMs for HMMER v3.4 (hmmer.org) using the hmmbuild command and then searched the resulting models against our genome data set using the nhmmer command separately for each spidroin class, terminal region and genome (Wheeler and Eddy 2013). The results were filtered to include only hits with the following characteristics: (1) *E*‐value < 10^−10^, (2) bit score > bias score and (3) hmm‐alignment length of at least 90% of the spidroin classes' pHMM length. Searching all spidroin classes separately, a spidroin terminal region may be found with several spidroin pHMMs. To assign the terminal region to the best fitting spidroin class, we compared all overlapping spidroin hits from all searches and assigned them to the classes according to the best hit, that is, the lowest *E*‐value. Since spidroins possess a structure of an N‐ and a C‐terminal domain, connected by a repetitive region, we matched those N‐terminal domains with the next C‐terminal domain on the same scaffold and combined them to full‐length spidroin genes. Since this might result in combining two independent hits, we filtered out all spidroin genes longer than 100 kbp. Most spidroin coding sequences known from the literature are around 10 kbp long (Zhao et al. 2006; Kono et al. 2019). This cut‐off should thus provide sufficient buffer to account for introns and longer than average genes but exclude unrealistic annotations. We then classified the spidroin hits based on the class assignment of both terminal domains. We used unclassified spidroin as a wildcard: Unclassified spidroin could match with any spidroin class. If there was discordance between the terminal domains' classes, we classified the gene as ‘discordant’.

We examined the evolutionary trajectory of spidroin class and copy numbers by reconstructing ancestral states and mapping them to a phylogeny using phytools' contMap command v2.0‐3 (Revell 2024) in R (R Core Team 2024). We created the phylogeny using 1859 arachnida_odb10 BUSCO genes, which were identified by the BUSCO software v5.4.5 and occurred in at least 95% of species (Seppey, Manni, and Zdobnov 2019). We aligned these sequences using MAFFT v7.520 and chose 100 random alignments to run ModelFinder via IQ‐tree v2.2.2.3 for substitution model selection (Kalyaanamoorthy et al. 2017; Minh et al. 2020). Then, we ran IQtree v2.2.2.3 with the suggested substitution model for the phylogenetic reconstruction on all alignments (Nakamura et al. 2018; Minh et al. 2020).

One peculiarity we encountered was that only a single N‐terminal domain (NTD) was identified for *R*. *nishihirai* although all known spidroins in spiders possess both a NTD and a CTD. To investigate this finding, we used publicly available transcriptome data of 12 species from five families belonging to two early diverging lineages Mygalomorphae and Mesothelae: *Phormictopus atrichomatus* (Foley et al. 2019), *Omothymus violaceopes* (Theraphosidae, previously labelled as *Lampropelma violaceopes* but the species was transferred to *Omothymus* by Gabriel and Sherwood 2019), *Atrax robustus* (Atracidae), *Atypus karschi* (Atypidae), *Heptathela yanbaruensis, Heptathela yakushimaensis*, *Heptathela kimurai*, *Ryuthela ishigakiensis*, *Ryuthela nishihirai* (2×), *Ryuthela sasakii* and *Liphistius murphyorum* (Liphistiidae, all Arakawa et al. 2022; accessions in Table 1). As the first step, we mapped the transcriptome reads of both available *R. nishihirai* RNA‐seq runs against our genome assembly using HISAT v2.2.1. to find out whether the hypothetical spidroin gets expressed. Since we found the gene being expressed, we searched for the first stop codon in the potential CTD‐region identified by the RNA data and extracted the 500 bp upstream. This should provide us with the potential CTD for this spidroin. Using online nucleotide Blast (Altschul et al. 1997) with settings for ‘Somewhat similar sequences (blastn)’, we were not able to find any similar sequence in NCBI. We then used an online version of AlphaFold v2.3.2 (available under https://colab.research.google.com/github/deepmind/alphafold/blob/main/notebooks/AlphaFold.ipynb?pli=1) to predict the structure of the CTD. Since we arbitrarily chose to extract 500 bp before the stop codon to include the C‐terminal region, we extracted the sequence that formed the alpha‐helical structures predicted by Alphafold to get the conserved CTD only. To investigate the presence of this C‐terminal domain in other early branching species, we assembled the downloaded RNA data using Trinity v2.15.1 (Grabherr et al. 2011). We then searched for the potential CTD sequence in the transcriptomes using HMMER v3.4's jackhmmer command (hmmer.org). We used the resulting hits to build a pHMM for the novel C‐terminal region and searched it against the genome assemblies as described before. The scripts and data used to create the spidroin annotation can be found under https://zenodo.org/doi/10.5281/zenodo.13711380.

### 2.7 Hox Genes

We also used pHMMs to identify and classify the hox genes in the spider genome data set. As training data, we used the Homeobox sequences of the hox genes identified in Harper et al. (2021). The reference data sets were aligned for each hox gene separately using MAFFT v7.520 and checked manually (Nakamura et al. 2018). From these alignments, we created pHMMs using HMMER v3.4's hmmbuild command (hmmer.org). Finally, we searched these profiles against the spider genome assemblies using HMMER v3.4's nhmmer command (Wheeler and Eddy 2013) separately for each assembly and hox gene. To filter for reliable hits, we included only those with an *E*‐value < 1 × 10^−20^. The identified homeoboxes were assigned to the hox class according to the lowest *E*‐value. For spiders, we expect two hox clusters, but only one to contain the *ftz* gene (Aase‐Remedios et al. 2023). Therefore, we used this characteristic to distinguish both clusters. We define the one that lost the *ftz* gene as Cluster B, and the other as Cluster A. The scripts and data used for the hox cluster annotation can be found under https://zenodo.org/doi/10.5281/zenodo.13711380. To check the gene order in the hox clusters, we manually removed those genome assemblies where the clusters were broken by a fragmented assembly (i.e., the breaks were directly at the end of a scaffold, Figure S19). To infer orthologous relationships, we assumed that genes of the same cluster represent orthologs. We used gggenomes (Hackl, Ankenbrand, and van Adrichem 2023) to generate Figure 4.

We manually determined the number of structural changes distinguishing each cluster from the structure we assume to be ancestral, that is, the structure present in the two earliest branching species *R. nishihirai* and *E. davidi*. We then reconstructed the ancestral states and mapped them to a phylogeny using phytools' contMap command v2.0‐3 (Revell 2024). We used the same phylogeny as for the spidroins.

## 3 Results

### 3.1 Karyotyping and Genome Size Estimation

**FIGURE 2:**
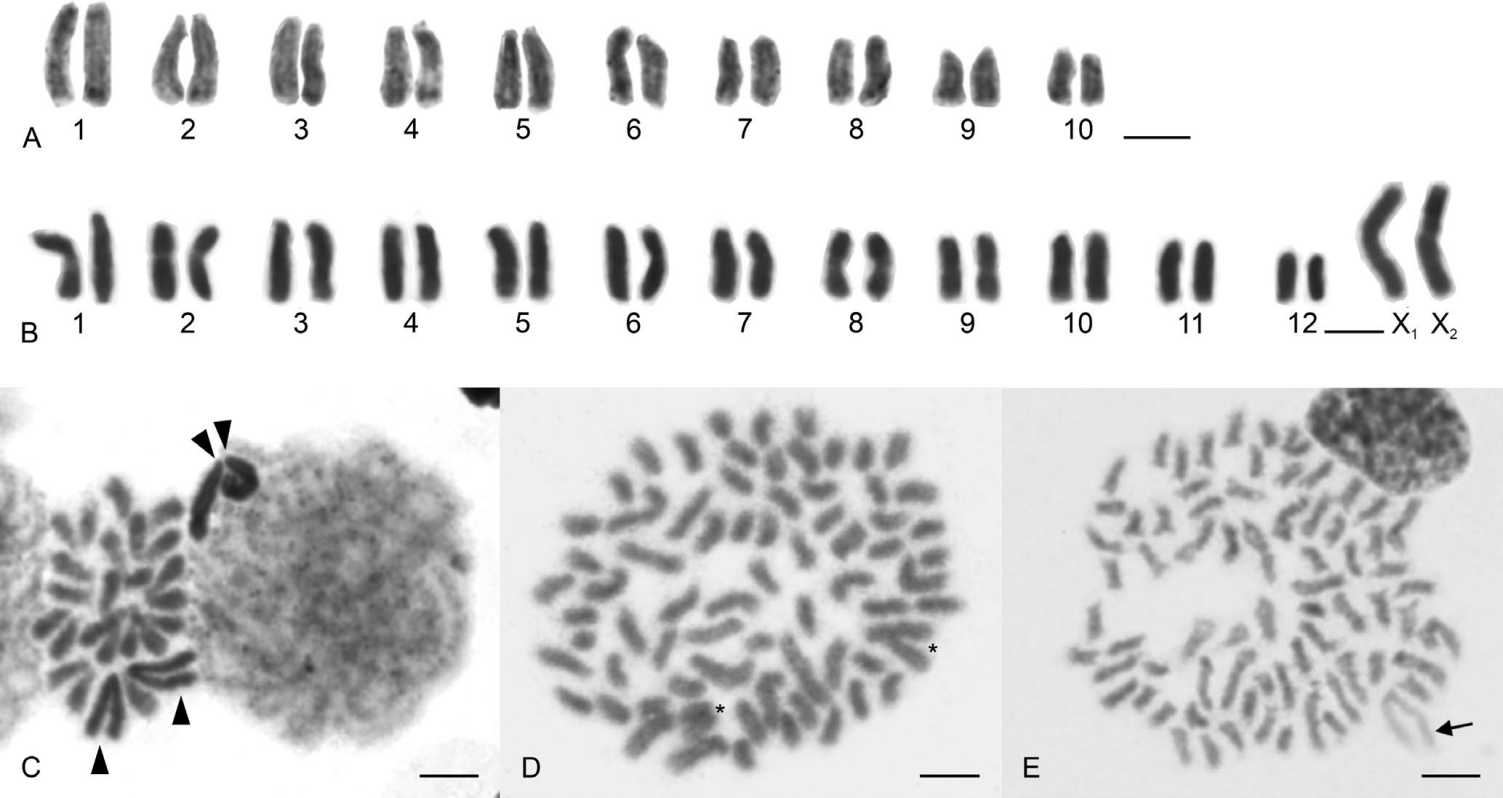
Chromosome data. (A) *U. plumipes*, female karyotype, based on mitotic metaphase. (B, C) *C. punctorium*, male. (B) Karyotype, based on mitotic metaphase. (C) Meiotic plates, metaphase II (left) and pachytene (right). Sex chromosomes are positively heteropycnotic, associated (metaphase II) or pair by centromeric regions (pachytene). (D, E) *R. nishihirai*, male, mitotic metaphases. (D) Sueyoshi, Okinawa Island (2*n* = 72). (E) Ryutan, Okinawa Island (2*n* = 71). Arrow—odd metacentric chromosome, arrowhead—X chromosome, asterisk—overlapping of two chromosomes. Scale bar = 5 μm. A detailed discussion can be found in Supporting Information S1.

The female karyotype of *U. plumipes* comprised 10 chromosome pairs (2*n* = 20), which were probably acrocentric. Lengths of chromosome pairs decreased gradually except for the last pair, which was considerably shorter than the penultimate pair (Figure 2A, Figure S20A). The length of the last pair was 54% of the length of the first pair (Figure S20A). Genome size (2C value) of the analysed female corresponds to 3.02 Gbp, and its genome had a GC‐content of 34.19%.

The male karyotype of *C. punctorium* consisted of 26 acrocentric chromosomes including two sex chromosomes, *X*_1_ and *X*_2_ (*X*_1_ *X*_2_0 system) (Figure 2B,C). Two chromosome pairs contained intercalar secondary constriction (pairs no 2, 8, Figure 2B). The longest pair was more than twice the length of the smallest pair (Figure S20B). Chromosome pair lengths decreased gradually except for the last pair, which was considerably shorter than the penultimate pair (Figure 2B, Figure S20B). Sex chromosomes were the longest elements of the karyotype, and had similar sizes (Figure 2B, Figure S20B). They were hyperspiralised and positively heteropyknotic (i.e., stained more intensively than the other chromosomes) during some meiotic phases (Figure 2C). Genome size (2C) of the studied male corresponded to 4.13 Gbp, and its genome had a GC‐content of 35.39%.

The male karyotype of *R. nishihirai* was composed of a high number of chromosomes, with acrocentric chromosomes predominating (Figure 2D,E). The karyotype from Sueyoshi was somewhat different from that from Ryutan. In the former, the karyotype was probably composed of 72 chromosomes (Figure 2D), in the latter of 71 chromosomes including the odd large metacentric chromosome (Figure 2E). A detailed discussion of the karyotyping results can be found in Supporting Information S1.

### 3.2 Genome Sequencing and Assembly

For *U. plumipes* and *C. punctorium*, we generated approx. 39 Gbp (26× read coverage) and 74 Gbp (36× read coverage) of CLR data, respectively. For *R. nishihirai*, sequencing yielded approximately 80 Gbp of HiFi‐Data (approx. 26× read coverage). The three final assemblies had a haploid sizes of: *C. punctorium* 2.6 Gbp (contig N50: 590 kbp); *U.plumipes* 1.5 Gbp (contig N50: 80 kbp); *R. nishihirai* 3.1 Gbp (contig N50 12, 9 Mbp, scaffold N50 75.5 Mbp, Table 2). BUSCO‐values showed quite different results: While the other two species yielded values above 95% Complete BUSCO genes, *U. plumipes* only contained 85.4% complete BUSCOs (Table 2). Merqury and Blobtools indicate that final assemblies contain few duplications and little contamination (Figure S1). Given that the assemblies are haploid, and have a high heterozygosity, the Merqury completeness values indicate contiguous assemblies: *C. punctorium* 79.05%; *U. plumipes* 76.05%; *R. nishihirai* 86.30%, after scaffolding 84.88%.

**TABLE 2:** BUSCO results and basic assembly statistics for all three assemblies.

Assembly	*Ryuthela nishihirai*	*Ryuthela nishihirai*	*U. plumipes*	*C. punctorium*
Level	Scaffold	Contig	Contig	Contig
# contigs	1713	2523	31,189	20,411
Largest contig	155,221,640	110,666,000	667,914	4,174,679
Total length	3,111,158,787	3,110,996,787	1,462,840,982	2,567,558,439
GC (%)	38.36	38.36	33.32	34.04
N50	75,489,856	12,891,142	79,661	589,820
N90	4,803,928	1,017,300	21,436	58,771
L50	16	56	5281	1161
L90	41	361	18,746	5920
*N*'s per 100 kbp	5.21	0	0	0
BUSCO				
Complete [%]	97.4		84.5	95.2
Single copy [%]	90		79.7	89.3
Multi copy [%]	7.4		4.8	5.9
Fragmented [%]	1		4.9	2
Missing [%]	1.6		10.6	2.8
Markers	2934		2934	2934

Repetitive sequences made up significant proportions of all three genomes. *Uloborus plumipes* had the lowest repeat content with 55.04%, and *R. nishihirai* had the highest with 69.09%. In all three genomes, interspersed elements represented the largest number of repeats, of which most were not classified. DNA‐transposons constitute a large portion among interspersed repeats in all three genomes. However, they had a lower proportion than retroelements and LINEs in *R. nishihirai*, which had far lower values in the other two species. All three species had few simple repeats and no satellites. However, in *R. nishihirai*, 11.4% of the genome comprised small RNA repeats, whereas in the other two, only a few repeats belonged to this class (Table 3).

**TABLE 3:** Repeat annotation for the assembled genomes.

	*R. nishihirai* (%)	*C. punctorium* (%)	*U. plumipes* (%)
Total	69.09	66.05	55.04
Retroelements	17.71	5.57	3.01
SINEs	1.45	0.63	0.23
Penelope	0.00	0.02	0.05
LINEs	15.08	1.81	1.36
LTR elements	1.18	3.13	1.43
DNA transposons	12.43	12.31	12.96
Rolling‐circles	0.04	2.86	1.64
Unclassified	28.51	44.09	35.55
Total interspersed repeats	58.65	61.99	51.57
Small RNA	11.40	0.78	0.24
Satellites	0.00	0.00	0.00
Simple repeats	0.35	0.84	1.50

### 3.3 Spidroins

As the first step in spidroin annotation, we searched for the terminal domains. As expected, we did not identify any terminal domains in the mite and scorpion genomes used as outgroups. We found full spidroin genes (i.e., having both a CTD and NTD) in all spider species, except two. The two exceptions were *R. nishihirai* and *D. silvatica* (Figure S21). For *R. nishihirai*, we only found a single; for *D. silvatica*, two N‐terminal domains. During manual inspection of the hits, it quickly became apparent that the spidroin sequences in *D. silvatica* were fragmented, as the spidroins terminated at the end of the contig without having a CTD. For *Ryuthela*, we found the potential spidroin to be complete and expressed. We extracted the 500 bp before the first stop codon in the C‐terminal region. A BLAST search against NCBI did not result in any meaningful hits. However, its predicted protein structure looked similar to other C‐terminal structures predicted by AlphaFold (e.g., Q2VLH4, Q2VLH3, A0A140DL57 in the AlphaFold Protein Structure Database, Jumper et al. 2021; Figure S22). The HMMER searches against the assembled transcriptomes of early branching spiders found the CTD only in four Mesothelae species.

We identified full spidroin sequences as having an NTD followed by a CTD. *Ryuthela nishihirai*, as the earliest branching spider, possessed a single, unclassified spidroin of 25,016 bp length. *Ectatosticta davidi* possessed a repertoire of five spidroins, comprising the classes MaSp, AcSp and Discordant. Most other species possessed several spidroin classes, each in multiple copies, with MaSp, MiSp and AcSp being the most frequent. The highest number of spidroin genes was found in *A. bruennichi* with 37 spidroin genes. Most spidroin classes had a median length below 10 kbp, except Flag and AgSp with approx. 20 kbp and 30 kbp, respectively. The shortest spidroin was a MaSp in *T. clavipes* and had a length of 1388 bp; the longest was a Flag found in *A*. *bruennichi* with a length of 74,112 bp (Figure S23).

### 3.4 Hox Genes

We found a single copy of the hox cluster and a free hox gene in the mites, and two copies in both scorpions and all spiders. The two copies are named Cluster A, B, respectively, and we distinguished them by the loss of *ftz* in Cluster B. Cluster B showed several inversions and rearrangements, which mostly involve two conserved subclusters (i.e., *lab*, *pb, Dfd* and *AbdB*, *AbdA*, *Ubx*, *Antp*, *Scr*). Cluster B reached lengths of several Mbp (e.g., > 40 Mbp in *D. plantarius*), usually separating the subclusters from each other (Figure 4). The *Hox3* gene of Cluster B was lost in all analysed spiders except *R, nishihirai*, *E. davidi* and *U. diversus*, but most species still had at least two copies, though the location of the *Hox3* genes varied considerably. Cluster A seems to have a highly conserved gene order and is quite compact (Figure 4). We observed three tandem duplications (*U. diversus*, *D. plantarius* and *H. graminicola*), four gene losses (*D. silvatica*, *H. graminicola* and both *Tetragnatha* species) and one case with larger restructuring of the entire cluster (*D. silvatica*). We observed six cases in which an additional gene copy of a hox gene was present on the same scaffold but clearly separated from the cluster by several Mbp (Figure 4).

## 4 Discussion

### 4.1 Genome Assemblies

In this study, we present genome assemblies of three species: *R.nishihirai*, *C. punctorium* and *U. plumipes*, each previously unsequenced. All genome sizes are consistent with the estimates by Flow Cytometry and the Kmer‐analysis (Table S1), indicating comprehensive assemblies.

The *U. plumipes* assembly represents the first of its species and second of the family Uloboridae, the only spider family to lose its venom apparatus, but not its venom (Peng et al. 2023). Our *C. punctorium* assembly represents the first genome sequenced for Cheiracanthiidae, only the second family sequenced from Dionycha, a clade representing well over a quarter of all known spider species (Gloor et al. 2024). Both genomes were assembled into contigs with high contiguity, making them valuable resources for evolutionary and comparative genomics studies. The *R. nishihirai* assembly was assembled to a higher completeness. 90% of the assembly length was anchored in the 41 largest scaffolds, which is close to the haploid number of this species (2*n* = 71–72), meaning that we achieved a near chromosome‐level assembly. Judging from the Hi‐C‐contact map and the Blobtools snail plots, we expect only very few chromosomes to be fragmented, indicating an extremely high contiguity and completeness (Figures S5 and S8). It is the first reference genome for suborder Mesothelae. The phylogenetic position of this suborder as sister to all other recent spiders, and *R. nishihirai*'s status as a ‘living fossil’ makes this assembly undoubtedly an extraordinary key resource enabling insights into early spider evolution. It will not just advance the research of this lineage but facilitate the study of evolution and diversity of spiders and their traits in general.

The repeat annotation revealed that all three assemblies comprise a high number of repeats. However, the repeat composition varied between *R. nishihirai* on the one hand, and *C. punctorium* and *U. plumipes* on the other hand (Table 3). Whereas the latter two assemblies contained only comparatively few retroelements, DNA‐transposons and LINEs, the *R. nishihirai* assembly contained these classes to a considerable fraction. Conversely, *R. nishihirai* had far fewer unclassified repeats. These divergences may indicate that repetitive elements have been quite active during spider evolution.

### 4.2 Spidroin and Hox Genes

**FIGURE 3:**
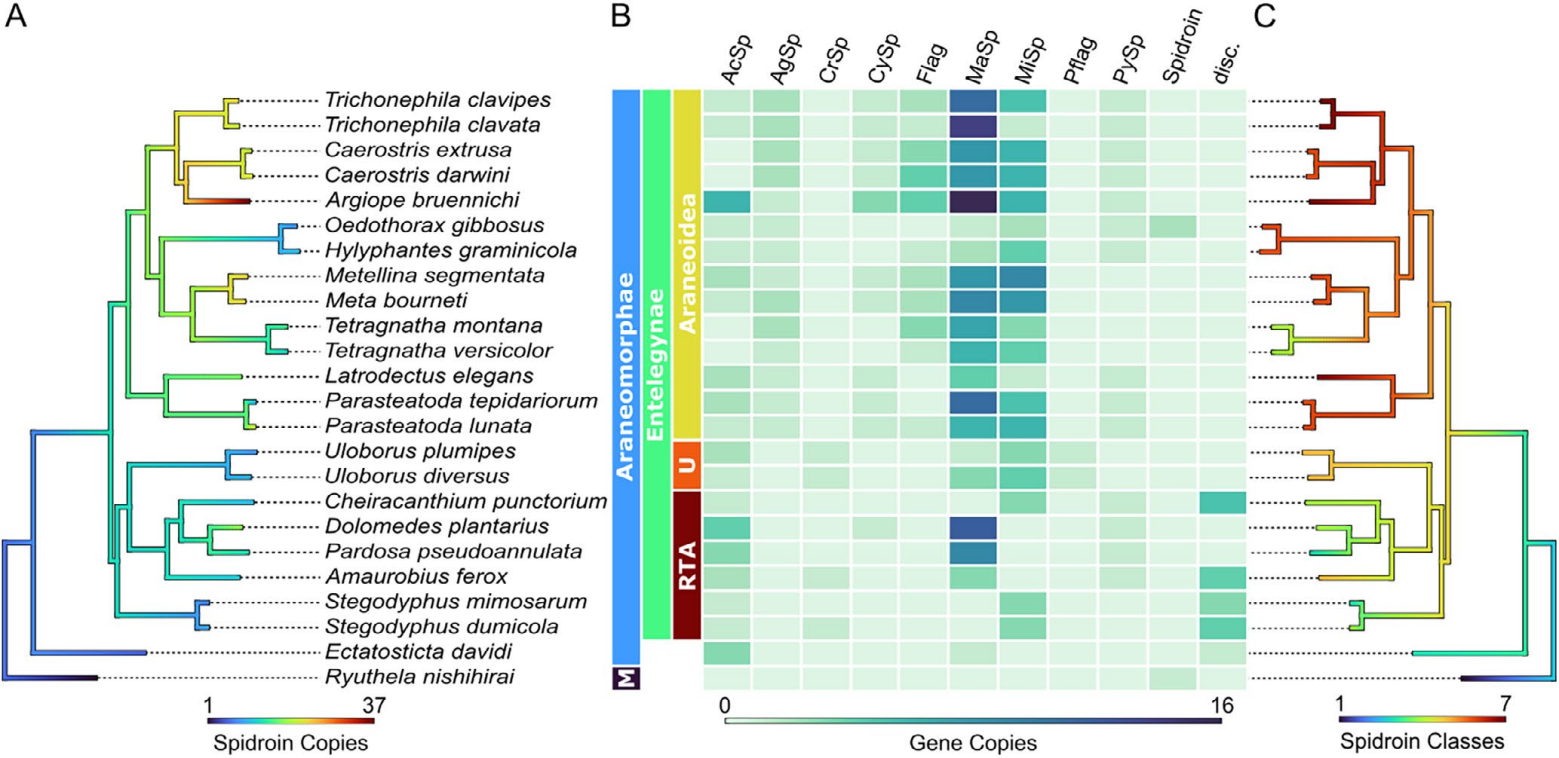
Distribution and expansion of different spidroin gene classes throughout the examined genomes. The two earliest branching lineages (*R. nishihirai*, *E. davidi*) possess comparatively few spidroin genes and classes, whereas Araneoidea seem to hold the highest spidroin diversity. The high number of discordant spidroins in the RTA clade might hint at hidden spidroin diversity (see discussion). (A) Observed and reconstructed number of spidroin copies. (B) Heatmap showing the copy numbers of each spidroin class per species. (C) Observed and reconstructed number of spidroin classes mapped to a phylogeny. AcSp, Aciniform; AgSp, Aggregate; CrSp, Cribellar; CySp, Cyllindrical; Flag, Flagelliform; MaSp, Major Ampullate; MiSp, Minor Ampullate; Pflag, Pseudoflagelliform; PySp, Pyriform; Spidroin, unclassified spidroin; disc., discordant (i.e., NTD and CTD were assigned to different classes); M, Mesothelae; U, Uloboridae; RTA, retrolateral tibial apophysis clade.

One key trait often associated with the success of spiders is their ability to use silk for various purposes, such as web building, protecting their offspring with a cocoon and even flying (ballooning) (Craig 2003; Foelix 2011). Our analysis mostly confirms the results by Arakawa et al. (2022), with Araneoidea holding the highest spidroin diversity (Figure 3). However, the analysis of full‐length spidroins revealed potential hidden spidroin diversity in the RTA‐clade and the so‐called UDOH grade (Uloboridae, Deinopidae, Oecobiidae and Hersiliidae). In this group, we found many discordant spidroins (i.e., such where NTD and CTD were assigned to different classes) in four out of eight species, showing that spidroin diversity might not be fully understood. In the three early branching species (*R. nishihirai*, *E. davidi* and *D. silvatica*), the spidroin diversity seemed relatively low, with only a few classes and copies. A similar pattern was already found by Arakawa et al. (2022) using a transcriptomic data set of terminal regions. As these three species represent the less diverse lineages, this pattern might support the frequently invoked hypothesis that spidroin diversification was a key development in spider evolution (e.g., Bond and Opell 1998; Craig 2003; Blackledge et al. 2009).

*Ryuthela nishihirai*, as a representative of Mesothelae, the suborder sister to all other recent spiders, had only a single, comparatively long (approx. 25 kbp) spidroin gene (Figure S23). Its spidroin possessed a hitherto unknown C‐terminal region with a predicted protein structure similar to other CTDs predicted using AlphaFold (e.g., Q2VLH4, Q2VLH3, A0A140DL57 in the AlphaFold Protein Structure Database, Jumper et al. 2021; Figure S22). Hence, we think it may be functionally equivalent to known CTDs. Finding only one spidroin gene supports the hypothesis that the last common ancestor of all recent spiders possessed a single spidroin gene as well. Although it is not clear how conserved the gene is, future in‐depth analyses of this spidroin promise unique insights into early spidroin evolution.

The hox cluster duplication within Arachnopulmonates represents an impactful evolutionary event, as hox genes have wide‐ranging regulatory functions in the development of bilaterians (e.g., Holland, Booth, and Bruford 2007; Larroux et al. 2007; Holland et al. 2017). Gene duplications have often been thought to be a potential driver of diversification, allowing sub‐ and neofunctionalisation of the gene copies (Ohno 1970; Lynch and Conery 2000). Therefore, the question arises, which role this duplication may have played in spider evolution. Cluster A seems highly conserved not only in structure but also in spatial extension, indicating conserved gene adjacency. The only exception is *D. silvatica*, which possessed a highly modified Cluster A. This finding represents a drastic change in the hox cluster, which relies heavily on its structure for spatial and temporal regulation (Ferrier 2019). Therefore, the altered structure may lead to a drastically altered functionality, potentially making it an interesting subject for studies of evolutionary development. Cluster B, on the other hand, shows considerable structural variation in araneomorphs often involving two subclusters. The subclusters were sometimes inverted and far from each other, though we never found the subclusters on different scaffolds. This means that genes within both subclusters have a conserved gene adjacency, whereas between subclusters the gene adjacency constraints seem relaxed, but not absent. Interestingly, *R. nishihirai*, *E. davidi* and *D. silvatica*, as well as *U. diversus*, retained a copy of the *Hox3* gene as part of Cluster B. In all remaining species, it was absent from Cluster B, indicating it might have lost its ancestral function there. However, all species except both *Tetragnatha* species still possess at least two copies of the *Hox3* gene. These findings indicate that the embryonic development of spiders is not yet fully understood, and additional evolutionary development studies of a higher diversity of species will probably yield even more variation.

**FIGURE 4:**
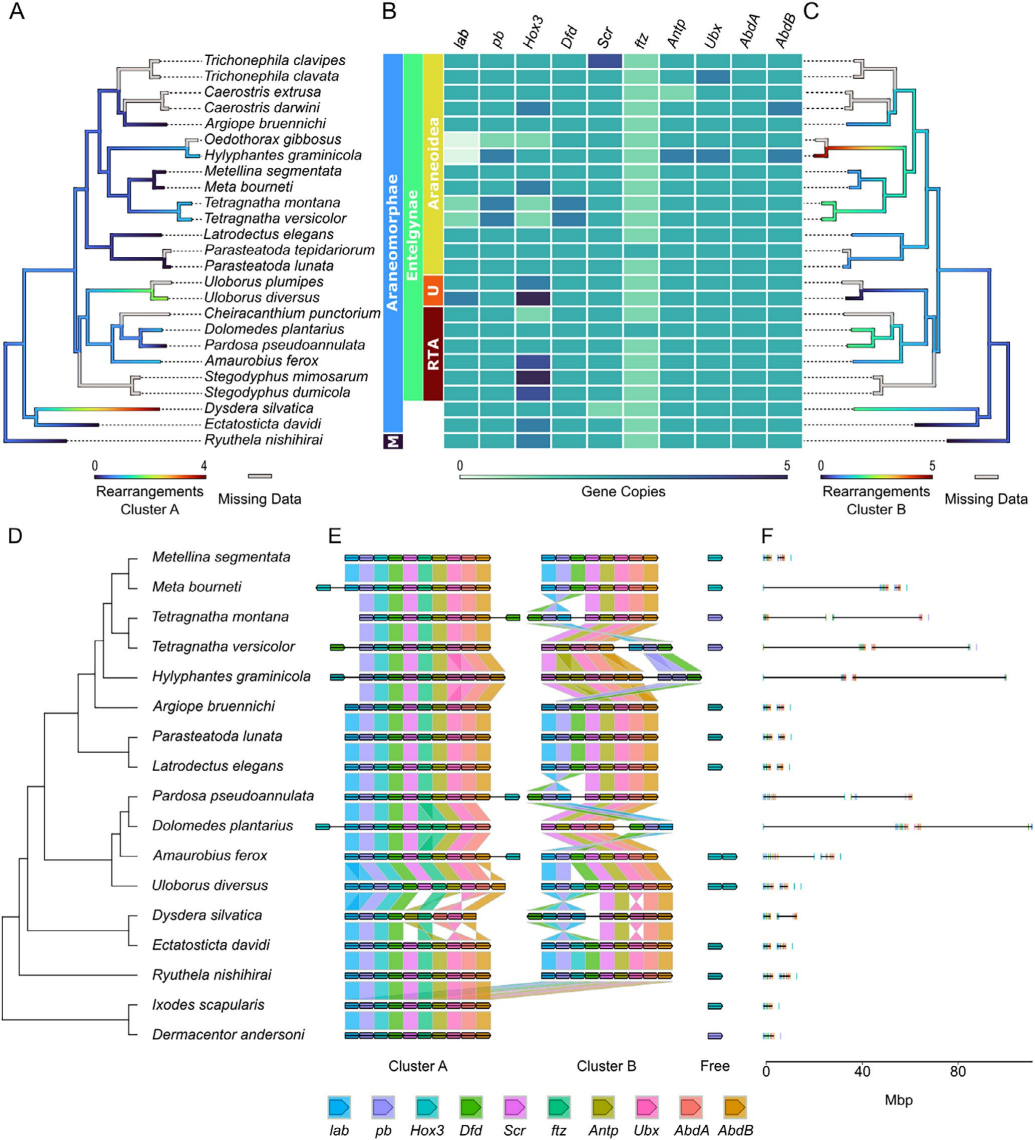
Identified Hox genes and the structural variation of the hox clusters. Most spiders possess two copies of each hox gene, except *ftz* for which one copy was lost in most spiders. Early branching species (*R. nishihirai*, *E. davidi*) possess conserved hox clusters, whereas younger species show considerable structural variation. This high structural plasticity mostly concerns Cluster B with with duplications, losses, inversions and rearrangements, Cluster A on the other hand seems highly conserved in most species (see text for details). (A) Number of observed and reconstructed rearrangements in hox cluster A. (B) Heatmap showing the copy number of each hox gene found per species. M, Mesothelae; RTA, retrolateral tibial apophysis clade; U, Uloboridae. (C) Number of observed and reconstructed rearrangements in hox cluster B. For species labelled with missing data, we could not reconstruct the structure of the hox clusters because of fragmented assemblies. (D) Cladogram of examined species. (E) Organisation of the hox clusters and their homologue relationships in different species. Larger gaps of > 5 Mbp are depicted by a blank black line. (F) Spatial distances between the hox genes from Panel E. The black vertical line indicates that the genes are on the same chromosome.

That Cluster B is less conserved than cluster A in numerous spiders was already demonstrated by Aase‐Remedios et al. (2023) using a limited set of spiders. However, new spider genomes from early diverging and comparably species‐poor lineages reveal an interesting pattern. Two of the three early branching spiders in our data set (*R. nishihirai* and *E. davidi*) retained a conserved structure of both hox clusters, despite > 350 My of divergence. The third species, *D. silvatica*, likely represents a special case as it is the only species in our analysis that acquired considerable restructuring of Cluster A. However, it is common ancestor with *E. davidi* likely had a conserved hox cluster structure (Figure 4). The species‐rich entelegyne spiders on the other hand accumulated considerable structural modifications of Cluster B in most species (Figure 4, Fernández et al. 2018; Howard et al. 2020). This raises the question of which role the duplication of the hox cluster and the relaxation of selective constraints may have played in spider evolution. Unfortunately, only a limited set of genomes were available at the time of analysis, missing important lineages such as the early branching Mygalomorphae. As increasing numbers of genomes across the whole spider tree of life become available, it should become easier to address this question.

## 5 Conclusion

We reported three novel genomes, each selected to cover previously unsequenced lineages, including the second family of the extraordinarily species‐rich Dionycha clade and the first reference genome for Mesothelae, a lineage of ‘living fossils’ and the suborder sister to all other living spiders. The unique phylogenetic positions of the genomes make them key resources to study spider evolution and diversity. Our analysis of spidroin genes and hox cluster diversity provides first insights into spidroin diversification and hox cluster duplication and restructuring, highlighting new questions for future studies.

## Author Contributions

Y.S.: designed research, performed research, analysed data, wrote the manuscript; A.B.H., M.F., T.K., L.P., and E.L.: performed research, revised the manuscript; J.K.: performed research, wrote the manuscript; T.L.A.: performed field work, revised the manuscript; C.M.: supervised research, revised the manuscript; H.K., S.P., and S.K.: designed research, wrote the paper, supervised research.

## Conflicts of Interest

The authors declare no conflicts of interest.

## Supporting information


**Data S1.**.

## Data Availability

All sequencing data and the assembled genomes are freely available on NCBI under Bio‐Project PRJNA1040011. The accessions of used genomes and transcriptome data are given in Table 1. The spidroin and hox gene annotations used can be found under https://zenodo.org/doi/10.5281/zenodo.13711380.
