# Supplementary material for: Three Novel Spider Genomes Unveil Spidroin Diversification and Hox Cluster Architecture: Ryuthela nishihirai (Liphistiidae), Uloborus plumipes (Uloboridae) and Cheiracanthium punctorium (Cheiracanthiidae)
Source: Mol Ecol Resour. 2024 Oct 22;25(1):e14038. doi: 10.1111/1755-0998.14038 (PMC11646306; doi:10.1111/1755-0998.14038)
Supplement: Supplementary file 1 — Data S1.. [file MEN-25-e14038-s001.docx]

**Supplemental Information for:**

**Three novel Spider Genomes unveil Spidroin Diversification and Hox Cluster Architecture: *Ryuthela nishihirai* (Liphistiidae), *Uloborus plumipes* (Uloboridae) and *Cheiracanthium punctorium* (Cheiracanthiidae)**

Yannis Schöneberg^1^, Tracy Lynn Audisio^7^, Alexander Ben Hamadou^8,9^, Martin Forman^2^, Jiří Král^2^, Tereza Kořínková^2^, Eva Líznarová^2,3^, Christoph Mayer^10^, Lenka Prokopcová^2^, Henrik Krehenwinkel^1^, Stefan Prost^4,5,6^, Susan Kennedy^1^

1 Department of Biogeography, Trier University, Universitätsring15, 54290, Trier, Germany

2 Laboratory of Arachnid Cytogenetics, Department of Genetics and Microbiology, Faculty of Sciences, Charles University, Viničná 5, 128 44 Prague 2, Czech Republic

3 Department of Botany and Zoology, Faculty of Science, Masaryk University, Kotlářská 267/2, 611 37, Brno, Czech Republic

4 Ecology and Genetics Research Unit, University of Oulu, Pentti Kaiteran katu 1, 90014 Oulu, Finland

5 South African National Biodiversity Institute, National Zoological Garden, 232 Boom Street, Pretoria 0002, South Africa.

6 Natural History Museum Vienna, Central Research Laboratories, Burgring 7, 1010 Vienna, Austria

7 Evolutionary Genomics Unit, Okinawa Institute of Science and Technology, 1919-1 Tancha, Onna-son, Kunigami-gun, Okinawa 904-0495, Japan

8 LOEWE -Centre for Translational Biodiversity Genomics (LOEWE-TBG), Senckenberganlage 25, Frankfurt Am Main, Germany

9 Senckenberg Forschungsinstitut und Naturmuseum, Frankfurt am Main, Germany

10 Centre for Molecular Biodiversity Research, Leibniz Institute for the Analysis of Biodiversity Change, Adenauerallee 127, 53113, Bonn, Germany

## Karyotyping - Discussion

The cytogenetic data on *Cheiracanthium punctorium* and *Uloborus plumipes* are consistent with existing knowledge on the karyotypes of entelegyne spiders. The karyotypes of these two species, as in most other entelegyne spiders (Kořínková & Král 2013), are composed of acrocentric chromosomes and include an unusual X**_1_**X**_2_**0 sex chromosome system, which is probably ancestral for entelegyne spiders (Král *et al.* 2006; Ávila Herrera *et al.* 2021).

So far, 12 species of the family Cheiracanthiidae belonging to three genera (*Cheiracanthium*, *Cheiramiona*, *Strotarchus*) have been karyotyped, including ten *Cheiracanthium* species (Araujo et al., 2024). Male karyotypes of the genus *Cheiracanthium* are diverse, ranging from 23 (*C. murinum*, Datta and Chatterjee, 1983) to 43 acrocentric chromosomes (*C. melanostomum* and *C. saraswatii*, Datta and Chatterjee, 1983; Srivastava and Shukla, 1986). However, most species possess 26 chromosomes and the X**_1_**X**_2_**0 system (Araujo et al., 2024), which is the karyotype we also observed in *C. punctorium* (this study). More detailed karyotype data were obtained for two species with 26 chromosomes, *C. mildei* and *C. pennyi* (Kumbıçak et al., 2014). Within *C. pennyi,* the lengths of chromosome pairs decrease gradually. In contrast, the penultimate pair is considerably shorter in *C. mildei* (Fig. 1C in Kumbıçak et al., 2014) and *C. punctorium,* which could be a synapomorphy of both species (this study, Figure S 20). However, unlike the species studied by Kumbıçak et al. (2014), sex chromosomes in *C. punctorium* are longer than the chromosome pairs (this study).

Concerning uloborids, chromosomes of eight species in three genera (*Miagrammopes*, *Octonoba*, *Uloborus*) have been studied, including four *Uloborus* species (Araujo et al., 2024). *Uloborus* species have diverse male karyotypes, ranging from 10 (*U. danolius*, Sharma and Parida, 1987) to 19 acrocentric chromosomes (*U. krishnae*, Datta and Chatterjee, 1983). Several sex chromosome systems are known for this genus, including X0, X**_1_**X**_2_**0, and X**_1_**X**_2_**X**_3_**0 systems (Araujo et al., 2024). The karyotype of *U. plumipes* consists of 18 acrocentric chromosomes, including the X**_1_**X**_2_**0 system (Mittal, 1970). This corresponds to the female karyotype of *U. plumipes* in our study, which consists of 20 chromosomes. In the case of the X**_1_**X**_2_**0 system, the female has two more X chromosomes than the male ((♂X_1_X_2_/♀X**_1_**X**_1_**X**_2_**X**_2_**).

Only a single Mesothelae, *Heptathela kimurai,* has been karyotyped so far (Suzuki, 1954). The karyotype of this species comprises a high number of acrocentric chromosomes. Suzuki reported two different diploid numbers for *H. kimurai* (80, Suzuki, 1949; approximately 96, Suzuki, 1954). The karyotype of *R. nishihirai* is also composed of a high number of chromosomes (2n = 71–72) with a predominance of acrocentric chromosomes (this study). Interestingly, the karyotypes of individuals from the Ryutan and Sueyoshi sites differ, which may reflect interspecific variation, as the taxonomy of *R. nishihirai* is still under discussion (Tanikawa, 2013; Xu et al., 2017). Alternatively, the differences may be due to chromosomal polymorphism. If so, the individual from Ryutan would be heterozygous for centric fusion.

## Data

### K-mer Analysis

Table S1: Results of K-mer analysis using GenomeScope and Flow Cytometry. Only the maximum values returned by the model are shown. See Supplement XXX for full table. All genome size estimations are consistent with the assembly sizes.

|  | ***Cheiracanthium***  ***punctorium*** | ***Uloborus***  ***plumipes*** | ***Ryuthela***  ***nishihirai*** |
| --- | --- | --- | --- |
| **Kmer-Analysis** |  |  |  |
| Homozygous [%] | 98.54 | 97.96 | 99.00 |
| Heterozygous [%] | 1.69 | 2.07 | 1.00 |
| Genome size (1C) [bp] | 2 380 083 385 | 1 242 582 647 | 2 322 884 680 |
| Repeat Length [bp] | 1 145 324 551 | 491 479 648 | 962 004 203 |
| Non-repetitive Length [bp] | 1 234 758 834 | 751 102 999 | 1 360 880 478 |
| Repeat Content [%] | 48.1 | 39.6 | 41.4 |
| Model Fit [%] | 98.94 | 98.48 | 97.00 |
| Read Error Rate [%] | 0.15 | 0.17 | 0 |
| **Flow Cytometry** |  |  |  |
| Genome size 2C [Gbp] | 4.13 | 3.02 |  |
| GC-content [%] | 35.39 | 34.19 |  |

Table S 2: Different parameter settings of the different wtdbg2 assemblies for *Cheiracanthium punctorium*. The quality metrics were generated using Quast and BUSCO with the arachnida_odb10 dataset (Gurevich *et al.* 2013; Seppey *et al.* 2019). The best results were achieved using wtdbg2 -p 16 -k 1 -K 2000 -AS 2 -e 3 -R -t 72 -L 3000 --aln-dovetail 2048 (third row in the table), as it had the highest BUSCO value.

| **Assembly** | **contigs** | **Largest contig** | **Length [Mbp]** | **N50** | **N90** | **L50** | **Comp.** | **Single Copy** | **Multi Copy** | **Frag.** | **Miss.** |
| --- | --- | --- | --- | --- | --- | --- | --- | --- | --- | --- | --- |
| wtdbg2 -L 5000 | 38 257 | 2 873 897 | 2 816 | 219 111 | 28 081 | 3 368 | 80.8 | 78.1 | 2.7 | 4 | 15.2 |
| wtdbg2 -p 17 -k 1 -K 2000 -AS 2 -e 3 -R \  -t 72 -L 3000 --aln-dovetail 2048 | 22 792 | 4 045 643 | 2 633 | 525 607 | 47 795 | 1 335 | 87.8 | 84.4 | 3.4 | 3.5 | 8.7 |
| wtdbg2 -p 16 -k 1 -K 2000 -AS 2 -e 3 -R \  -t 72 -L 3000 --aln-dovetail 2048 | 20 802 | 4 204 746 | 2 606 | 591 384 | 55 911 | 1 174 | 89 | 85.4 | 3.6 | 3.2 | 7.8 |
| wtdbg2 -p 17 -k 1 -K 2000 -AS 2 -e 3 -R \  -t 72 -L 3000 --aln-dovetail 2048 | 22 771 | 4 045 643 | 2 632 | 526 888 | 47 734 | 1 334 | 87.7 | 84.3 | 3.4 | 3.5 | 8.8 |

Table S 3: Different parameter settings of the different wtdbg2 assemblies for Uloborus plumipes. The quality metrics were generated using Quast and BUSCO with the arachnida_odb10 dataset (Gurevich *et al.* 2013; Seppey *et al.* 2019). The best assembly was achieved using wtdbg2 -AS 2 -K 2000 -e 2 -p 16 -k 1 --align-dovetail 3072 (last row in the table), as it had the fewest BUSCO genes missing.

| **Assembly** | **# contigs** | **Largest contig** | **Tot. Length [Mbp]** | **N50** | **N90** | **L50** | **Comp.** | **Single Copy** | **Multi Copy** | **Frag.** | **Miss.** |
| --- | --- | --- | --- | --- | --- | --- | --- | --- | --- | --- | --- |
| wtdbg2 default | 42 719 | 596 929 | 1 488 | 60 018 | 15 074 | 7 014 | 41.6% | 41.0% | 0.6% | 4.7% | 53.7% |
| wtdbg2 -S 2 | 46 182 | 850 962 | 1 596 | 60 674 | 14 994 | 7 428 | 44.4% | 43.5% | 0.9% | 5.1% | 50.5% |
| wtdbg2 -AS 2 -K 2000 | 43 246 | 759 652 | 1 622 | 68 181 | 16 189 | 6 637 | 47.5% | 46.5% | 1.0% | 5.1% | 47.4% |
| wtdbg2 -AS 2 -K 2000 -e 2 | 43 241 | 759 651 | 1 622 | 68 164 | 16 189 | 6 637 | 47.6% | 46.6% | 1.0% | 5.1% | 47.3% |
| wtdbg2 -AS 2 -K 2000 -e 2 -p 17 -k 1 | 42 115 | 744 082 | 1 619 | 70 779 | 16 641 | 6 404 | 47.4% | 46.5% | 0.9% | 5.0% | 47.6% |
| wtdbg2 -AS 2 -K 2000 -e 2 -p 16 -k 1 | 41 365 | 1 115 940 | 1 620 | 72 372 | 16 775 | 6 214 | 48.3% | 47.6% | 0.7% | 4.9% | 46.8% |
| wtdbg2 -AS 2 -K 2000 -e 2 -p 17 -k 0 | 41 589 | 732 805 | 1 625 | 72 242 | 16 742 | 6 281 | 47.9% | 47.4% | 0.5% | 5.2% | 46.9% |
| wtdbg2 -AS 2 -K 2000 -e 2 -p 16 -k 1 \  --align-dovetail 512 | 40 697 | 672 494 | 1 613 | 74 411 | 16 852 | 6 076 | 48.2% | 47.5% | 0.7% | 5.1% | 46.7% |
| wtdbg2 -AS 2 -K 2000 -e 2 -p 16 -k 1 \  --align-dovetail 1024 | 39 083 | 746 412 | 1 590 | 77 925 | 17 319 | 5 713 | 48.8% | 48.0% | 0.8% | 5.0% | 46.2% |
| wtdbg2 -AS 2 -K 2000 -e 2 -p 16 -k 1 \  --align-dovetail 2048 | 36 477 | 748 174 | 1 556 | 83 256 | 18 355 | 5 229 | 49.0% | 48.3% | 0.7% | 4.9% | 46.1% |
| wtdbg2 -AS 2 -K 2000 -e 2 -p 16 -k 1 \  --align-dovetail 3072 | 35 092 | 725 904 | 1 538 | 86 954 | 18 870 | 5 013 | 48.9% | 48.0% | 0.9% | 5.2% | 45.9% |

### *Ryuthela nishihirai*.


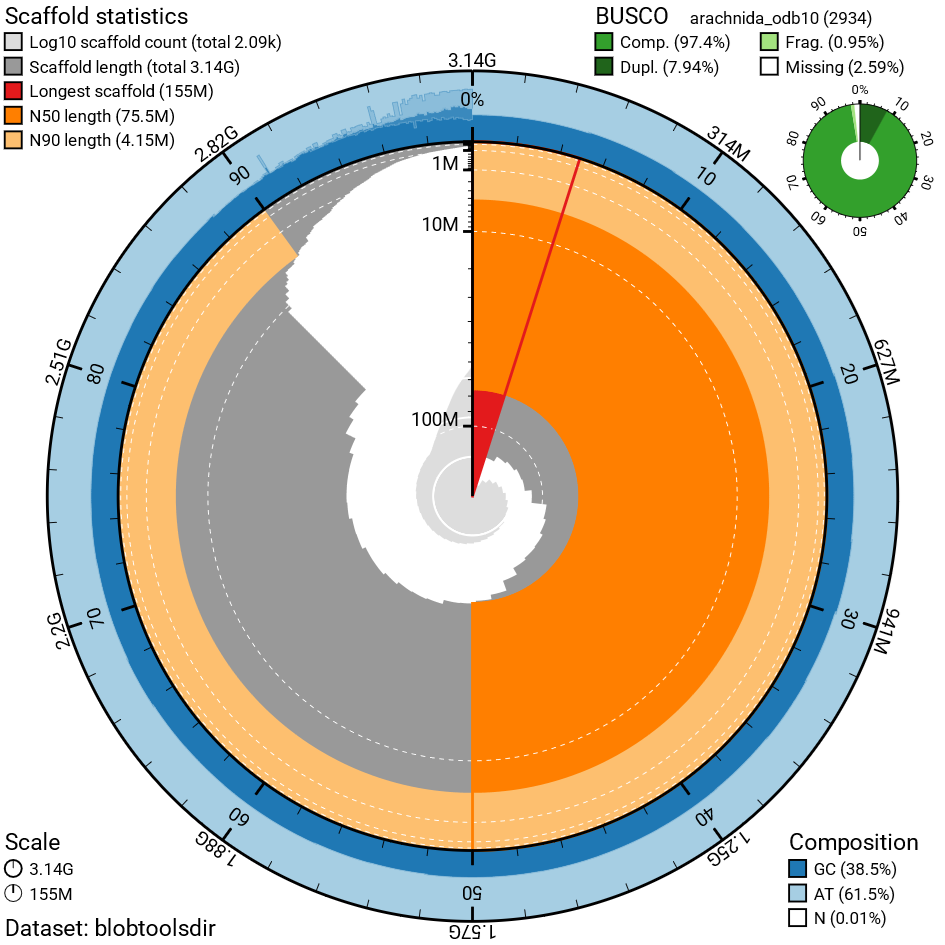


Figure S 1.Snail plot for the *R. nishihirai assembly*, before filtering contaminations. It shows the assembly contiguity, BUSCO-scores, GC-composition. The inner spiral depicts the scaffold lengths on a radial scale. The N50 and N90 values are shown in dark and light orange, respectively, the longest scaffold is coloured red. The blue rim shows the GC/AT content across the genome. The assembly is near chromosome level.


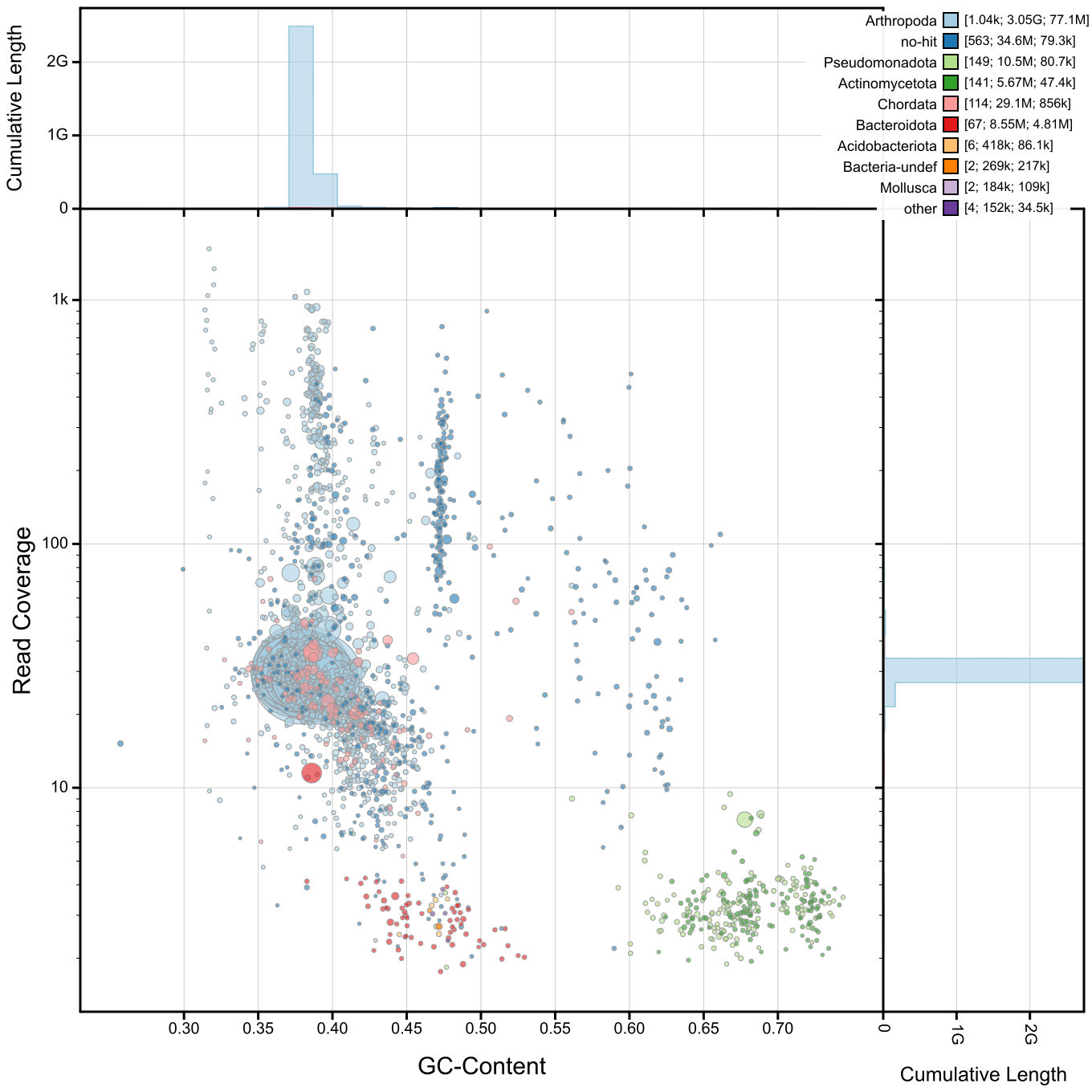


Figure S 2:Blobtoolkit scatter plot showing potential contamination for *R. nishihirai*, before filtering contaminations. There is contamination visible: The redish cluster (mainly Bacteriodota, and undefined Bacteria) and the greenish cluster (mainly Actinomycetota). In the following step, we removed this contamination.


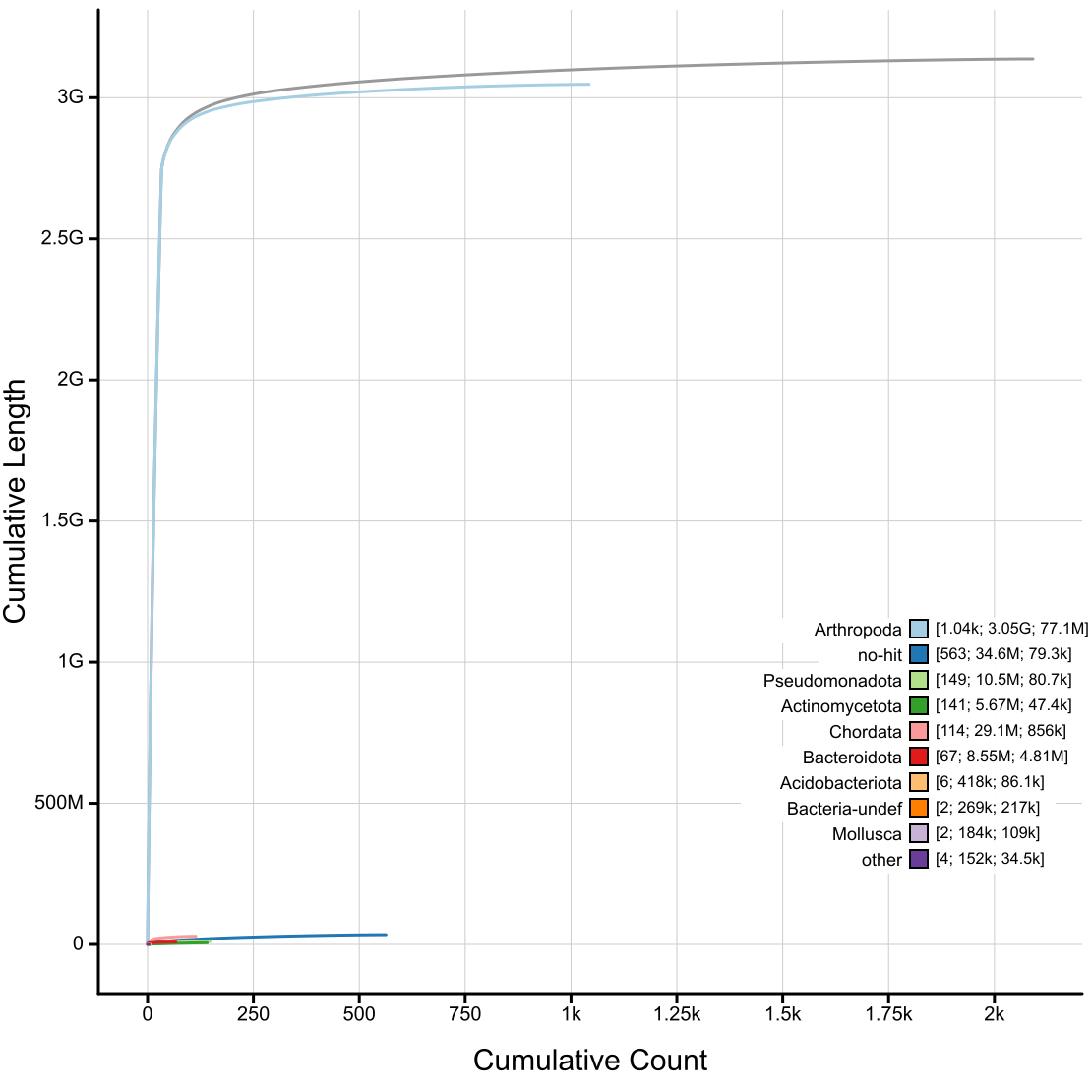


Figure S 3: Plot of the cumulative assembly length and the respective taxonomic assignment for *R. nishihirai*. This plot indicates the presence of contamination, mainly Bacteriodota, undefined Bacteria and Actinomycetota. These contaminations were filtered in the next step.


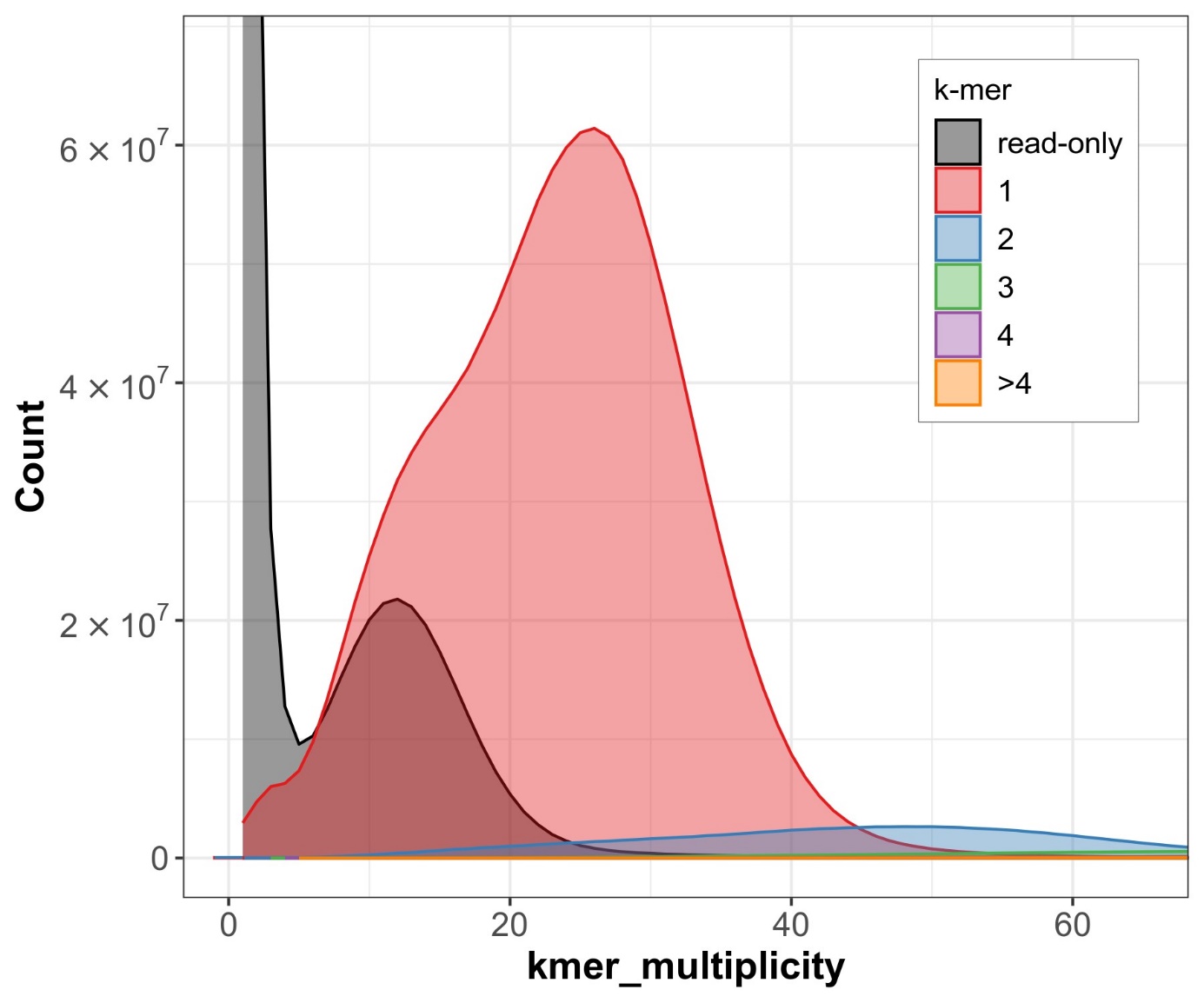


Figure S 4: Merqury plot used to quality control the *Ryuthela nishihirai* assembly before scaffolding. It indicates a high completeness and does not show hints for duplicated haploid contigs.

####
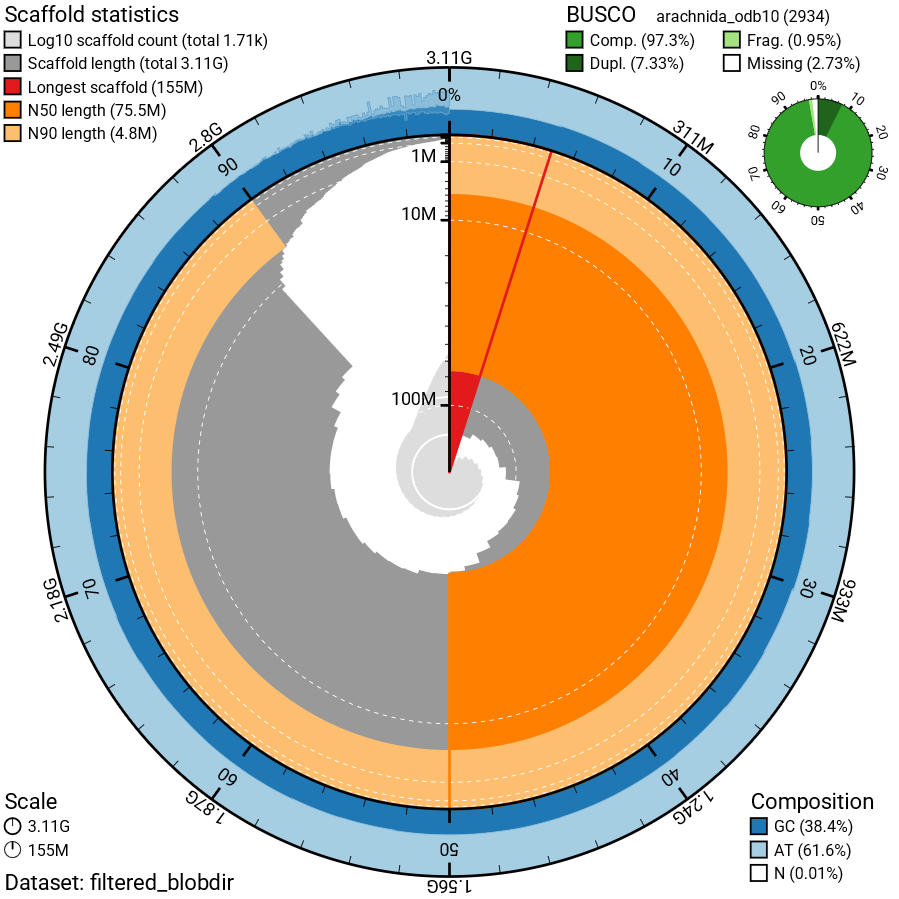


Figure S 5:Blobtoolkit Snail plot for the *R. nishihirai assembly*, after filtering contaminations. It shows the assembly contiguity, BUSCO-scores, GC-composition. The inner spiral depicts the scaffold lengths on a radial scale. The N50 and N90 values are shown in dark and light orange, respectively, the longest scaffold is coloured red. The blue rim shows the GC/AT content across the genome. The assembly is near chromosome level.

####
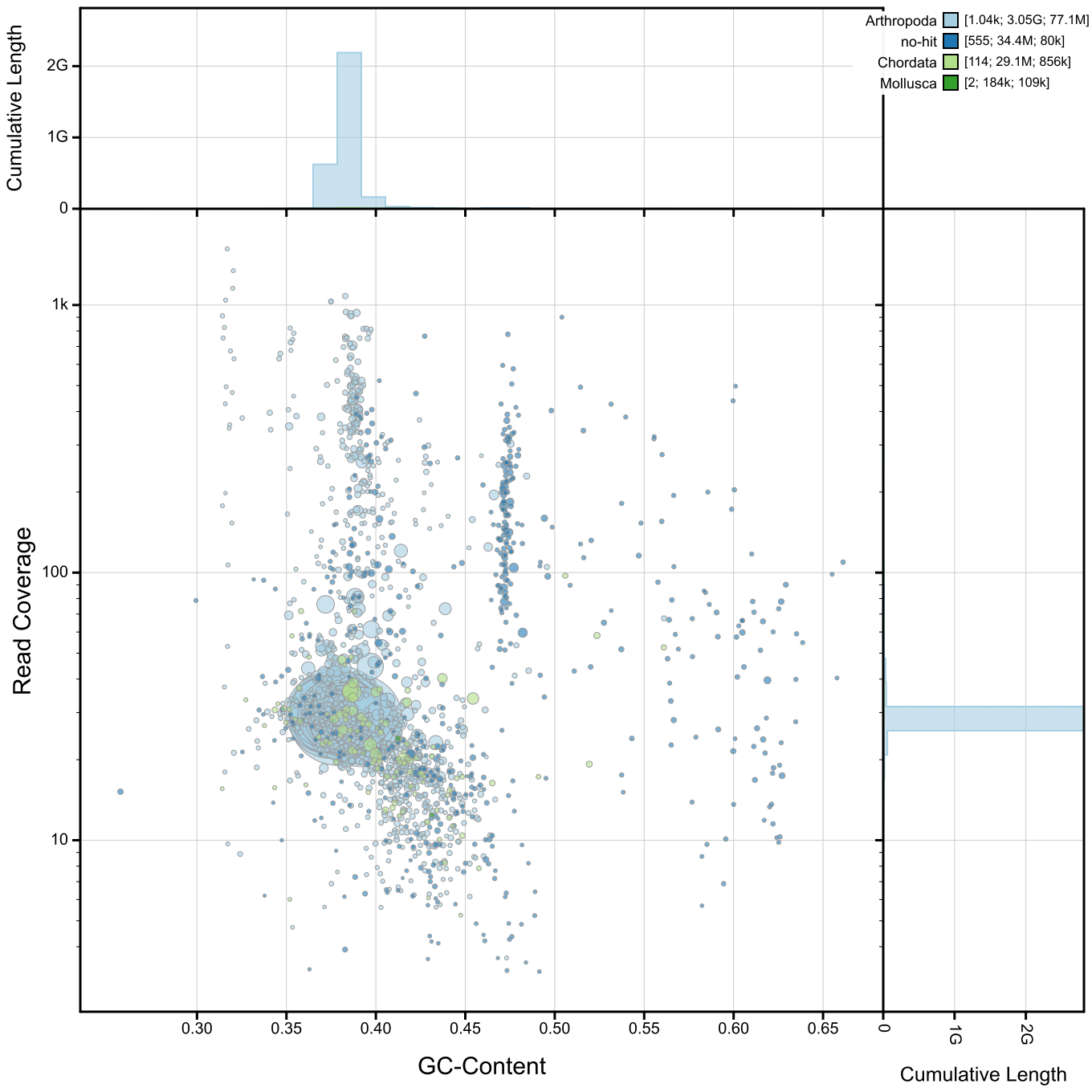


Figure S 6:Blobtoolkit scatter plot showing potential contamination for *R. nishihirai*, after filtering contaminations. There is no contamination visible: The redish cluster (mainly Bacteriodota, undefined Bacteria) and the greenish cluster (mainly Actinomycetota) in Figure S 2 both were filtered out.

####
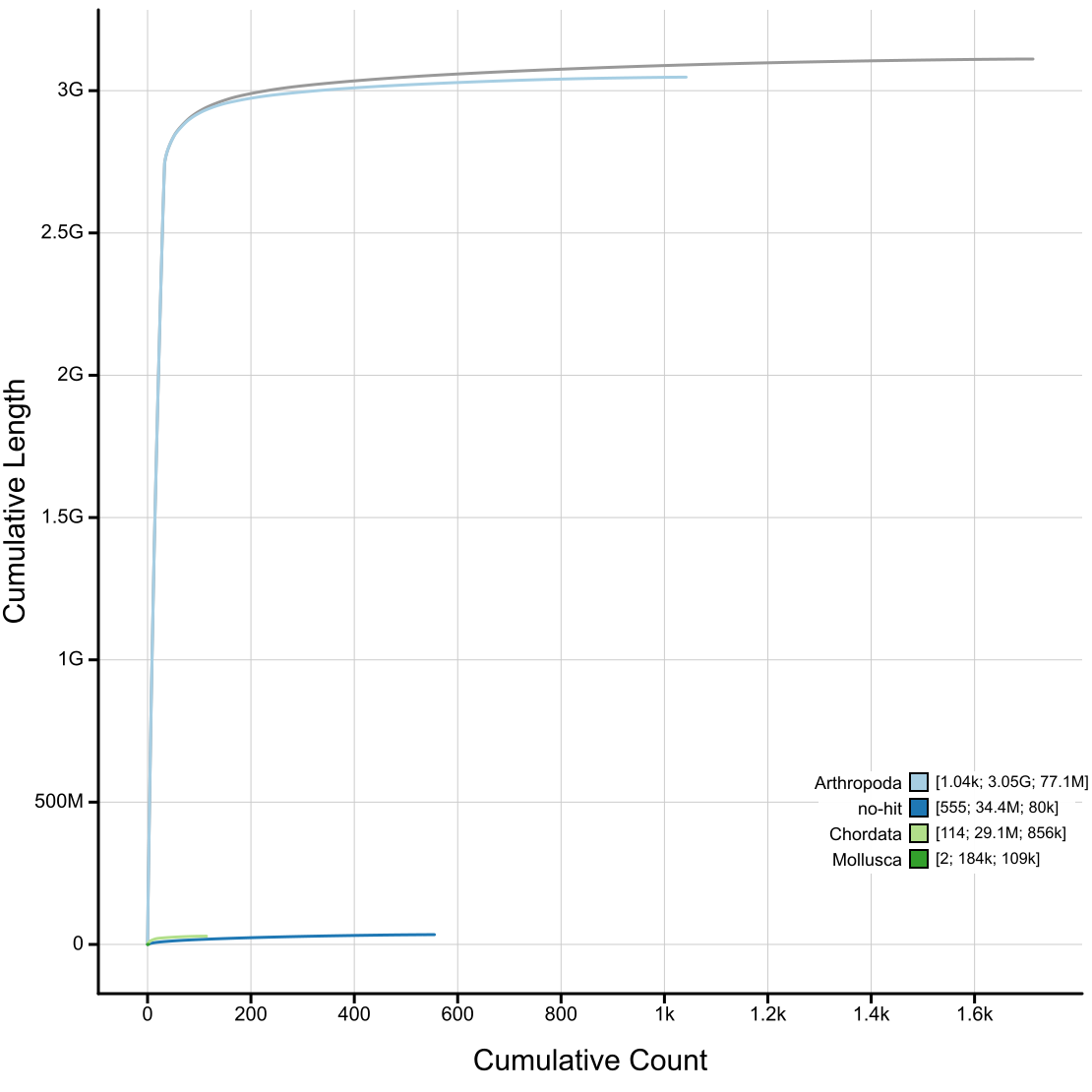


Figure S 7: Plot of the cumulative assembly length and the respective taxonomic assignment for *R. nishihirai* after removing contamination. This plot shows there is only marginal contamination in the assembly.


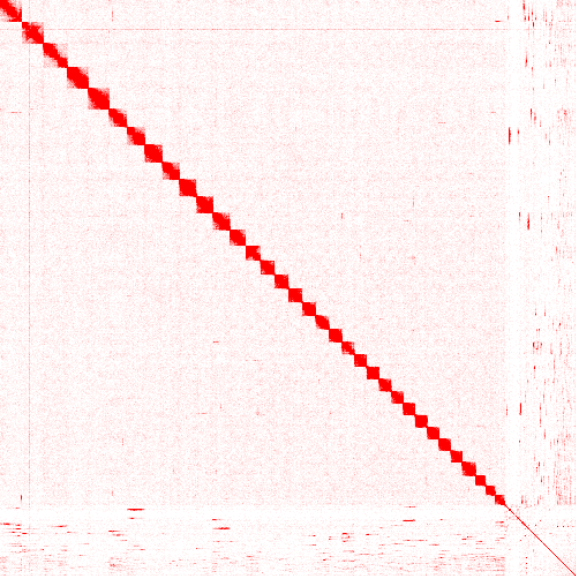


Figure S 8: HiC-contact map for *R. nishihirai* showing high contiguity. 90% of the assembly was scaffolded into 41 scaffolds, indicating a near chromosome level assembly.


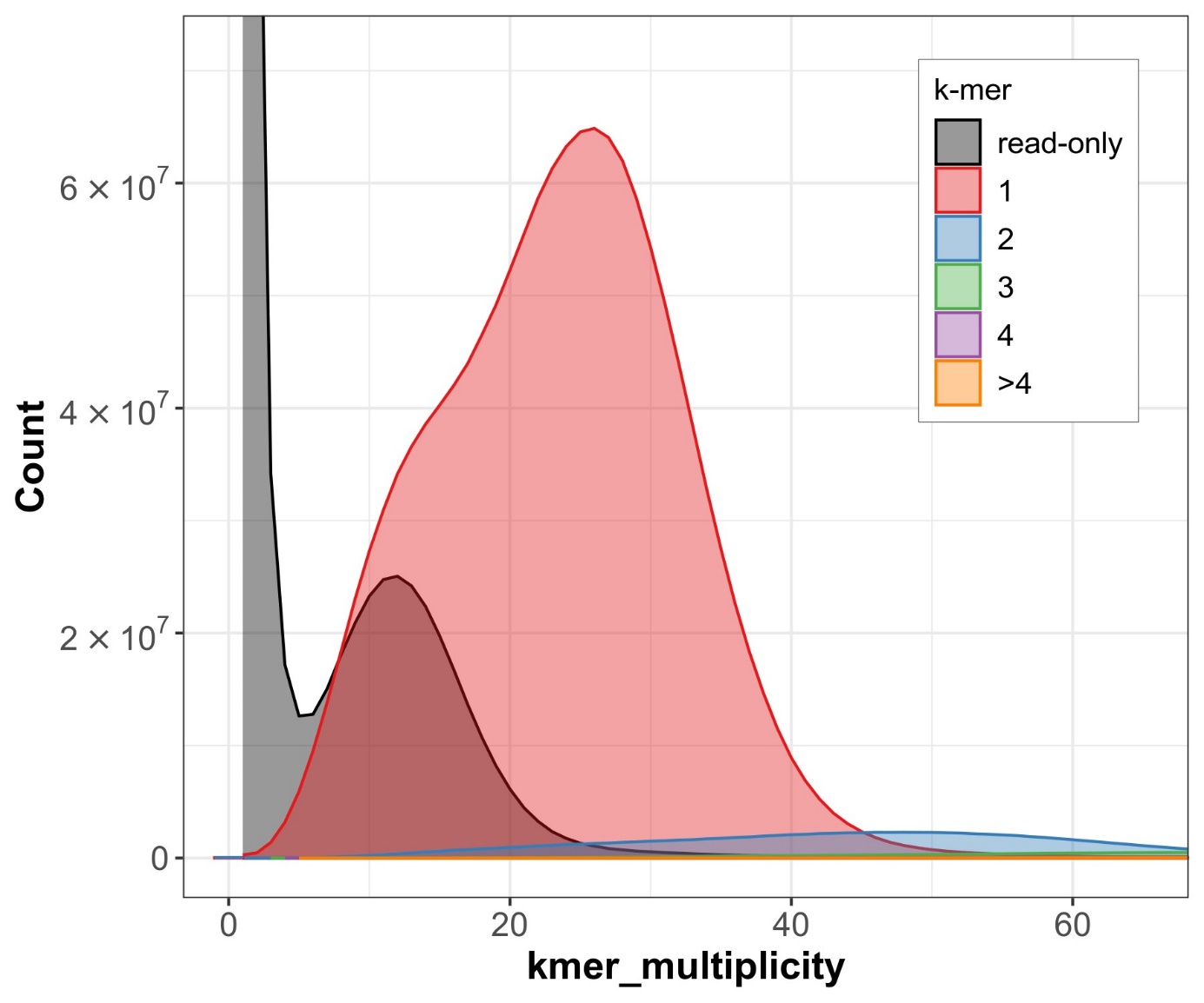


Figure S 9: Merqury plot used to quality control the *Ryuthela nishihirai* assembly after scaffolding and gapfilling. It indicates a high completeness and does not show hints for duplicated haploid contigs.

### *Cheiracanthium punctorium*


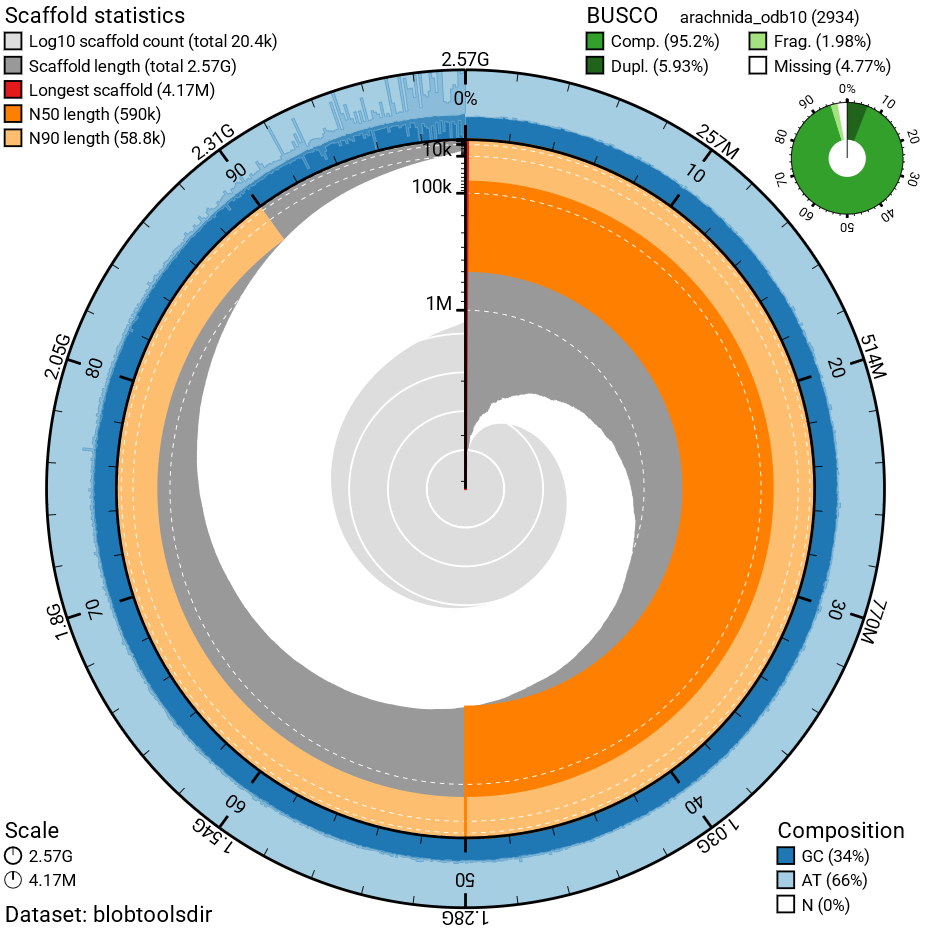


Figure S 10: Snail plot for the *C. punctorium* assembly, before filtering contaminations. It shows the assembly contiguity, BUSCO-scores, GC-composition. The inner spiral depicts the scaffold lengths on a radial scale. The N50 and N90 values are shown in dark and light orange, respectively, the longest scaffold is coloured red. The blue rim shows the GC/AT content across the genome. The assembly is near chromosome level.


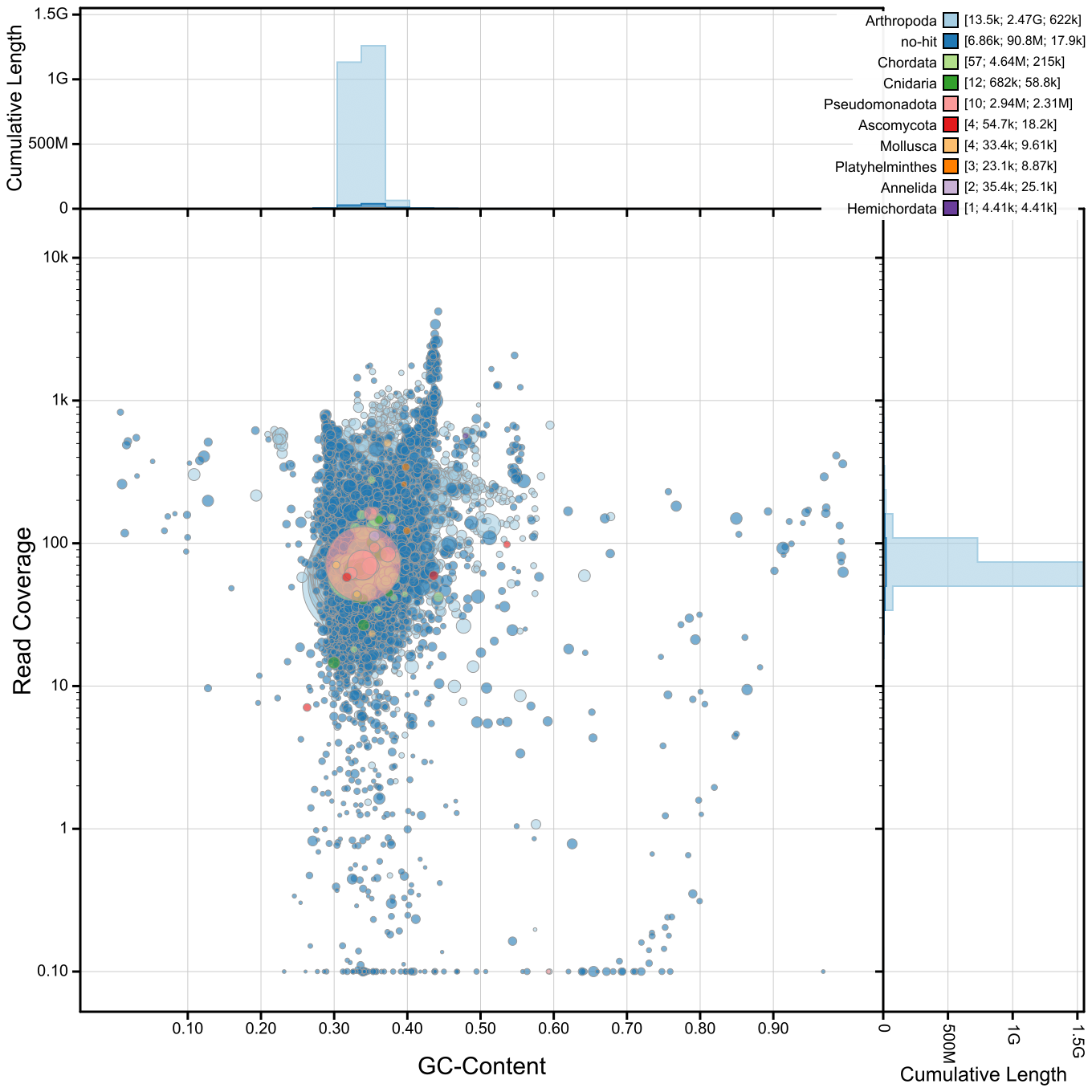


Figure S 11: Blobtoolkit scatter plot showing potential contamination for *C. punctorium*. The assembly does not hold much contamination and contigs assigned to other taxa are likely false positives.


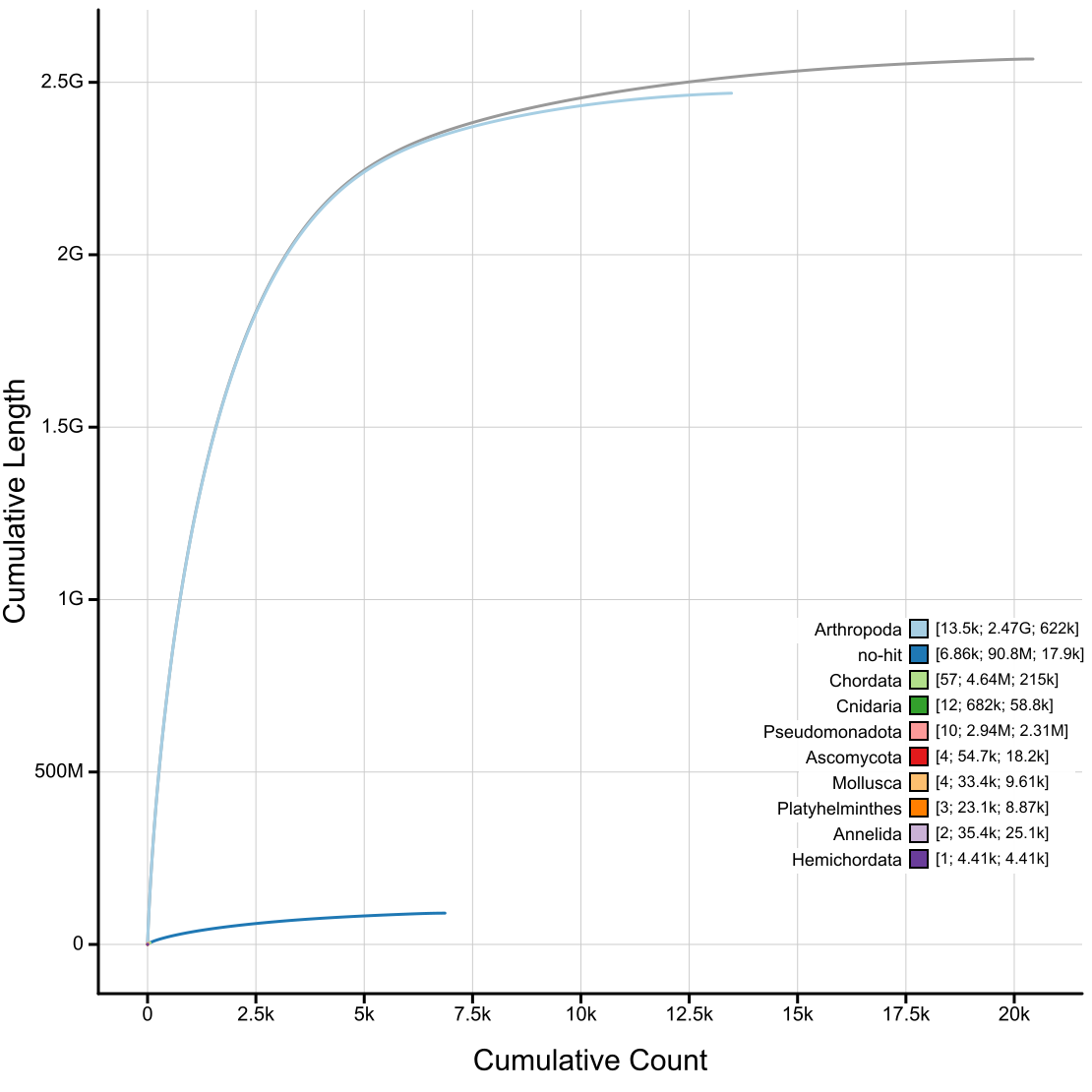


Figure S 12: Plot of the cumulative assembly length and the respective taxonomic assignment for *C. punctorium*. This plot shows there is only marginal contamination in the assembly.


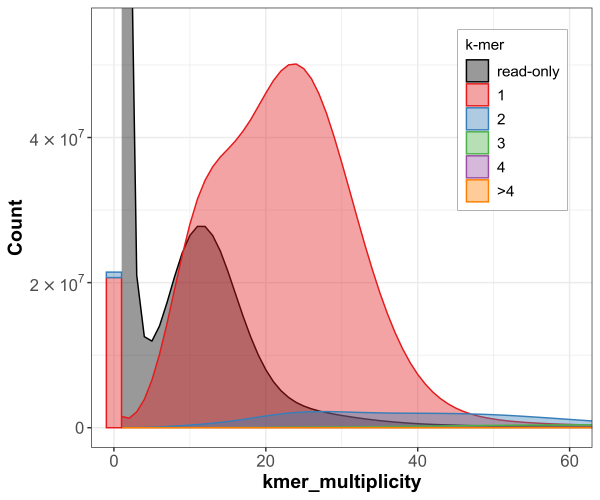


Figure S 13: Merqury plot used to quality control the *Cheiracanthium punctorium* assembly. It indicates a high completeness and does not show hints for duplicated haploid contigs.

### *Uloborus plumipes*


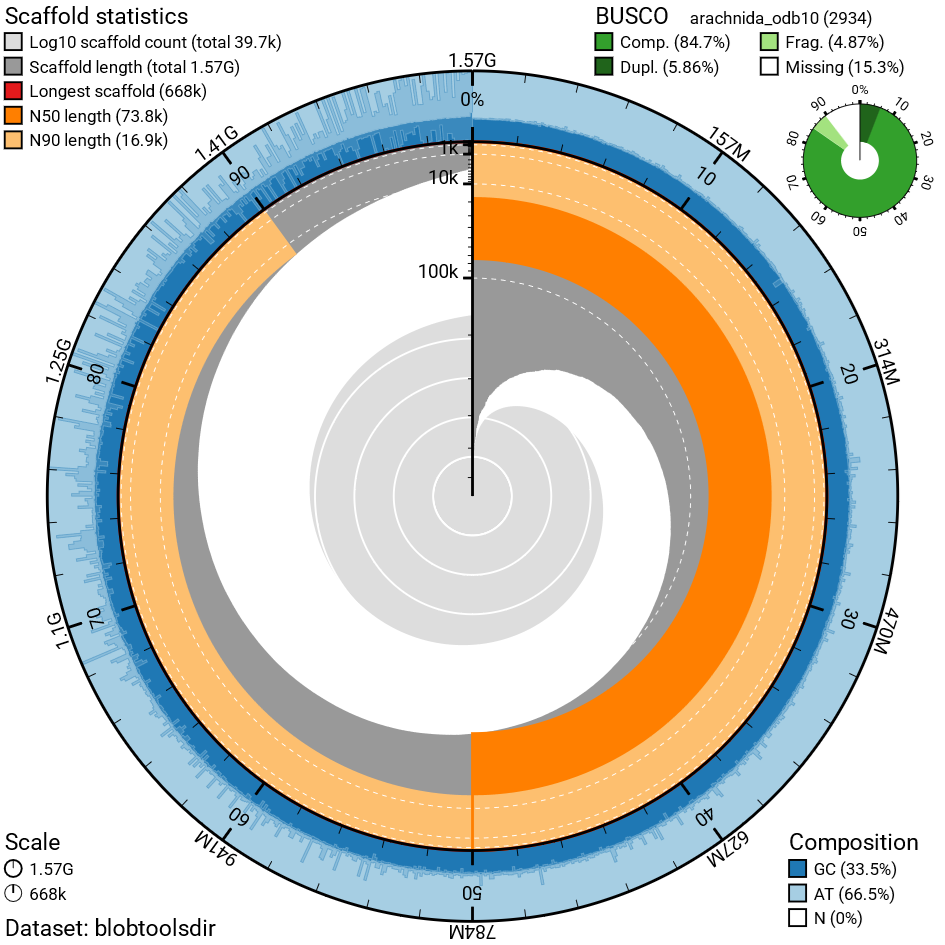


Figure S 14: Snail plot for the *U. plumipes* assembly, before filtering contaminations. It shows the assembly contiguity, BUSCO-scores, GC-composition. The inner spiral depicts the scaffold lengths on a radial scale. The N50 and N90 values are shown in dark and light orange, respectively, the longest scaffold is coloured red. The blue rim shows the GC/AT content across the genome. The assembly is near chromosome level.

*
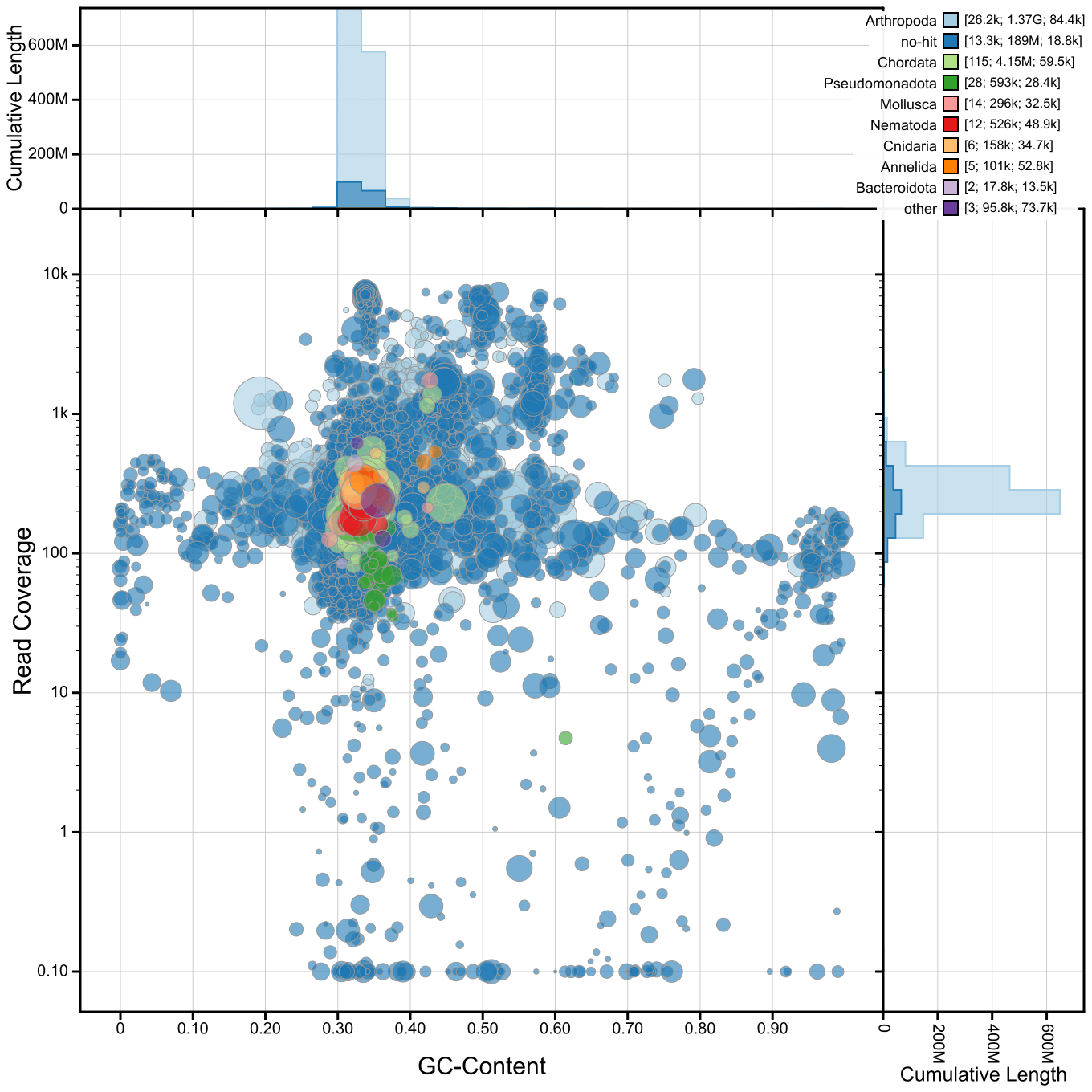
*

Figure S 15: Blobtoolkit scatter plot showing potential contamination for *U. plumipes*. The assembly does not hold much contamination and contigs assigned to other taxa are likely false positives.


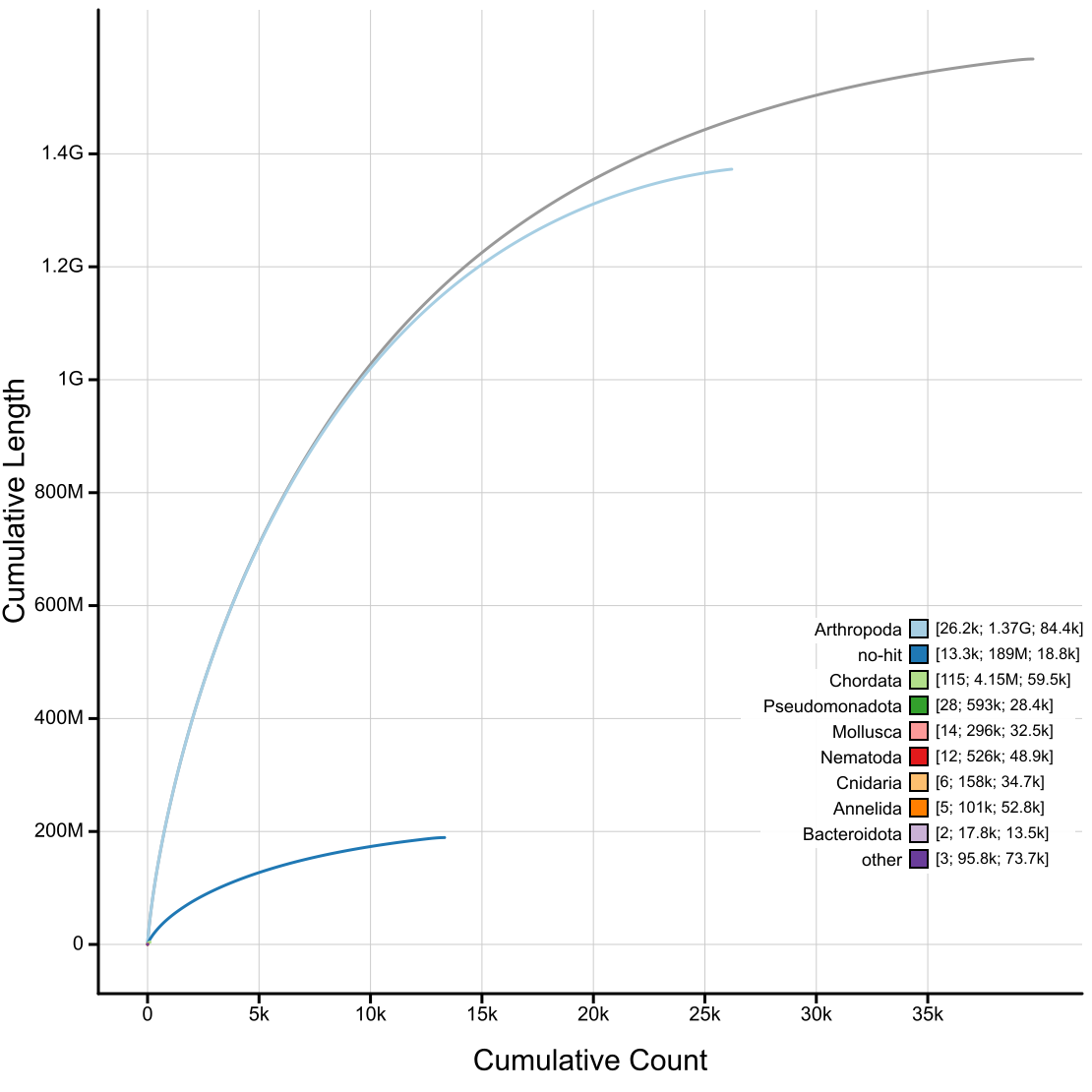


Figure S 16: Plot of the cumulative assembly length and the respective taxonomic assignment for *C. punctorium*. This plot shows there is only marginal contamination in the assembly.


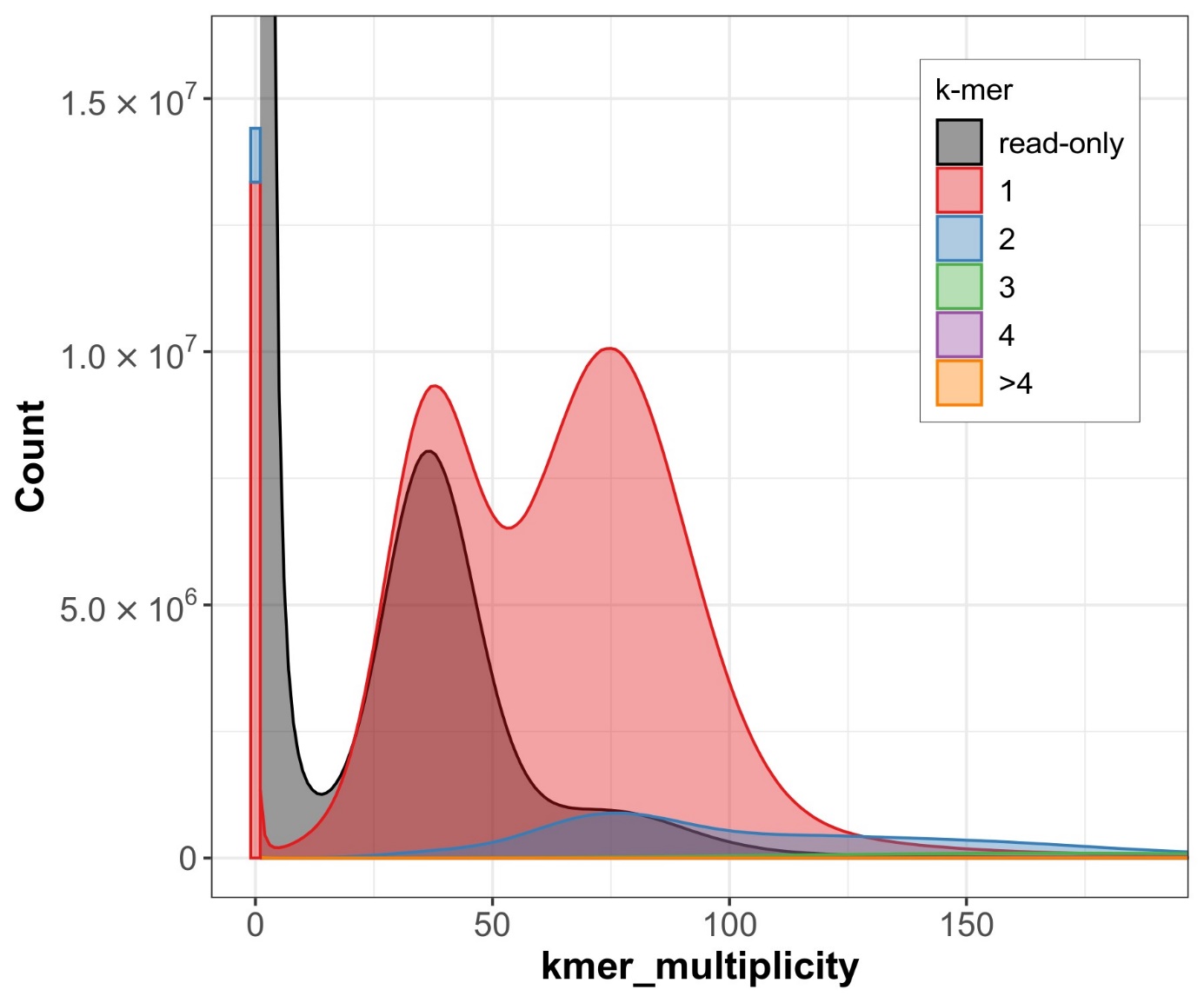


Figure S 17:Merqury plot used to quality control the Uloborus plumipes assembly before purging duplicated haploid contings. The hump of kmers which are found 2 times in the assembly and have a coverage of approx. 75X indicate duplicated haploid contigs. Therefore, we purged these using purge_haplotigs.


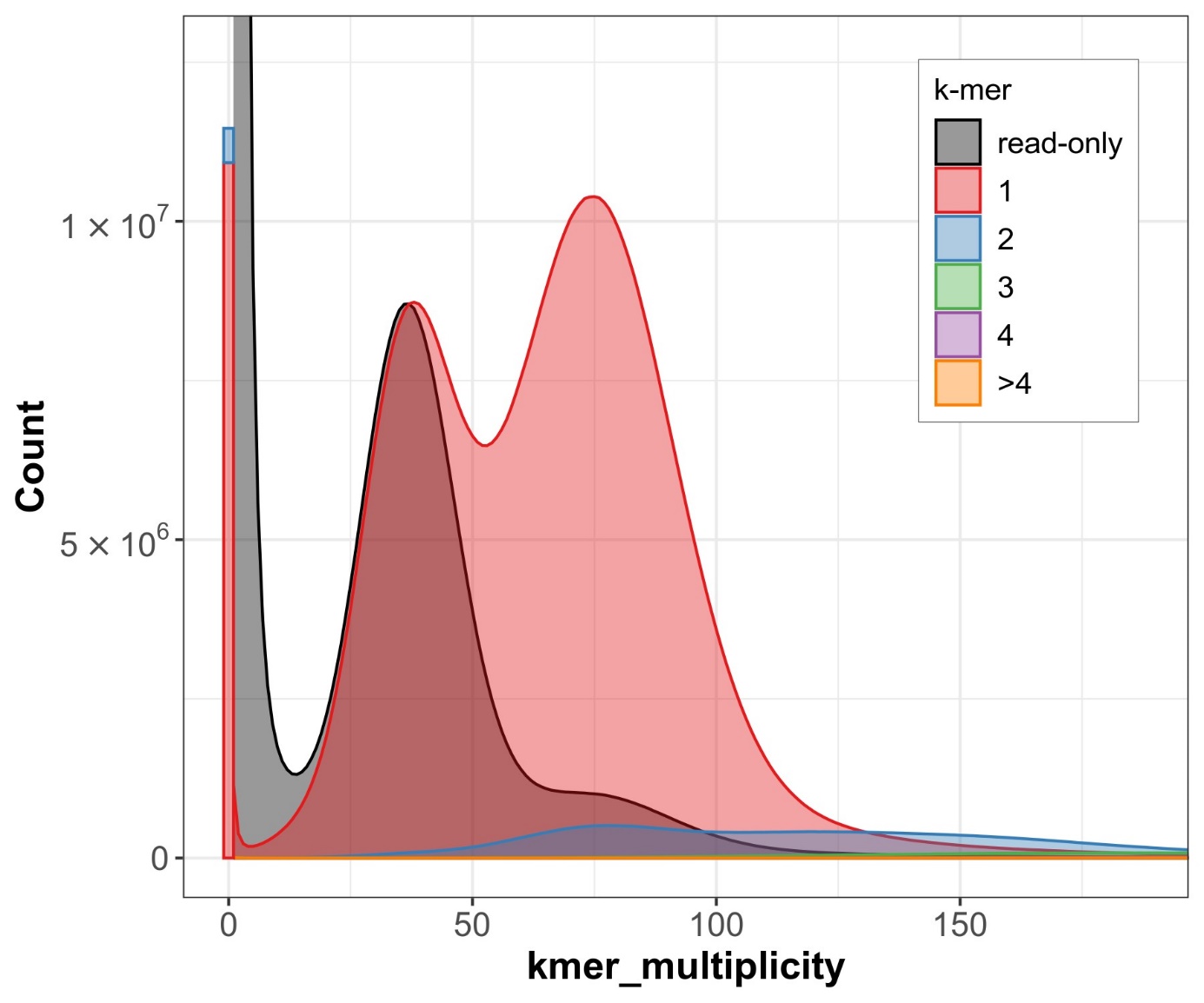


Figure S 18: Merqury plot used to quality control the *Uloborus plumipes* assembly after purging duplicated haplotigs. Purging haplotigs visibly reduced the number of duplicated haploid contigs.


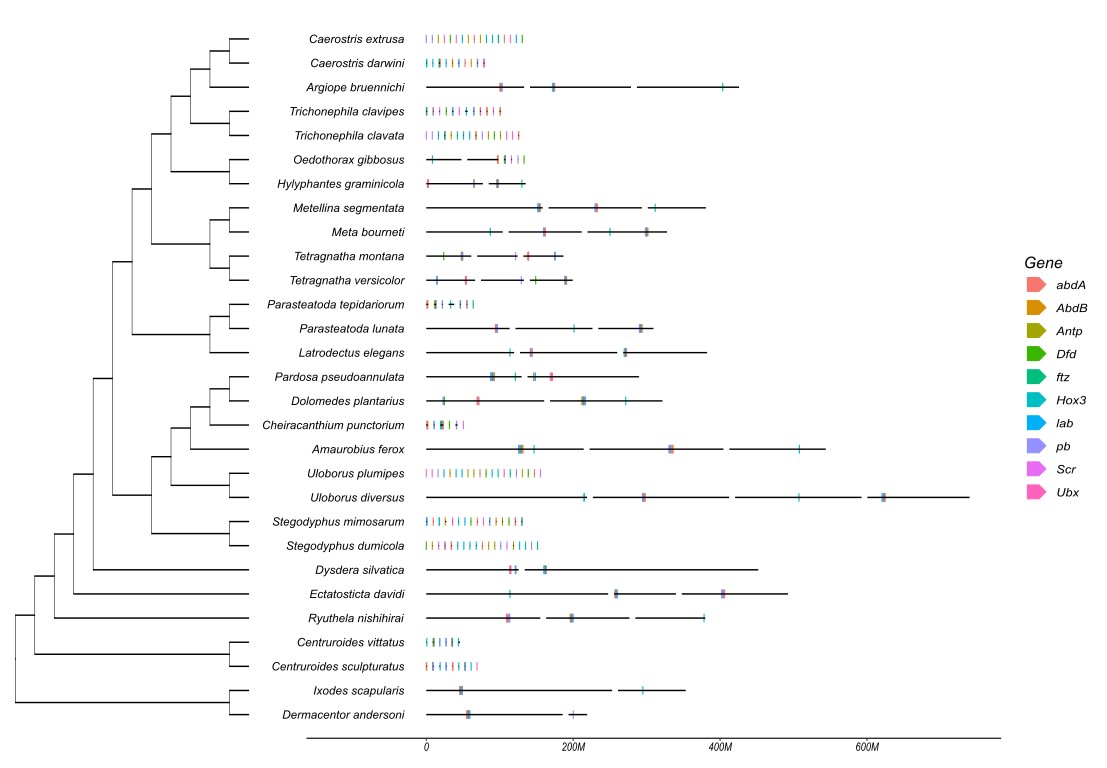


Figure S 19: Distribution of hox genes on the scaffolds. Vertical black lines indicate hox genes, and horizontal lines indicate the scaffold. The black horizontal line represents the scaffold and the vertical lines the positions of the annotated hox genes on the scaffold. We excluded those genomes from structural analyses, where the hox cluster was apparently fragmented.


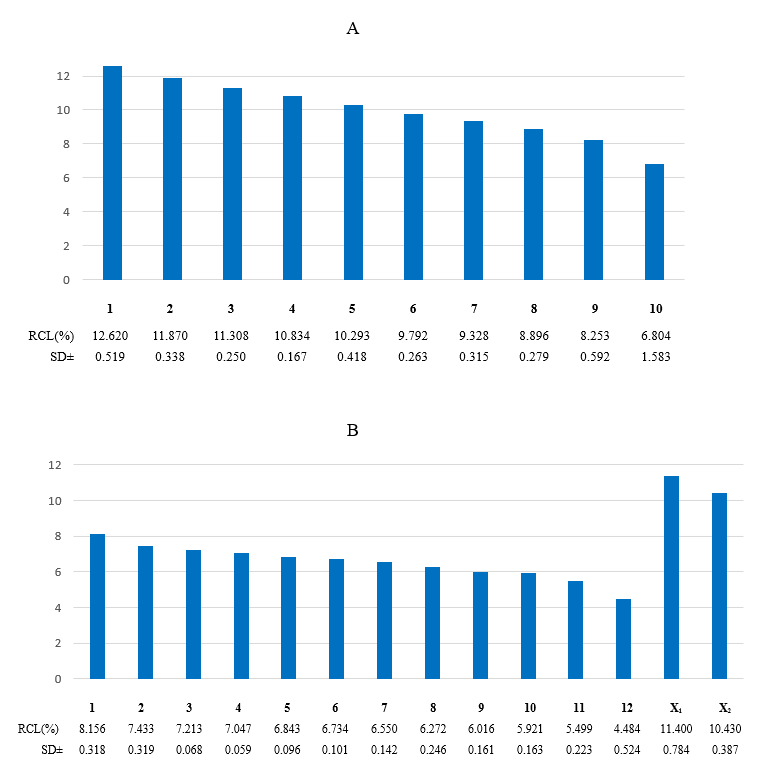


Figure S 20: Analysed entelegyne spiders, haploid chromosomal ideograms. On the X-axis, the chromosomes representing the individual chromosome pairs are indicated by numbers, as well as the sex chromosomes X_1_ and X_2_ are listed. On the Y-axis, the relative chromosome length (RCL) is indicated. The RCLs are also given numerically (including standard deviation, SD), below the X-axis. (**A**) *Uloborus plumipes*, female, based on mitotic metaphases (n = 5). (**B**) *Cheiracanthium punctorium*, based on plates formed by two sister metaphases II (n = 4).


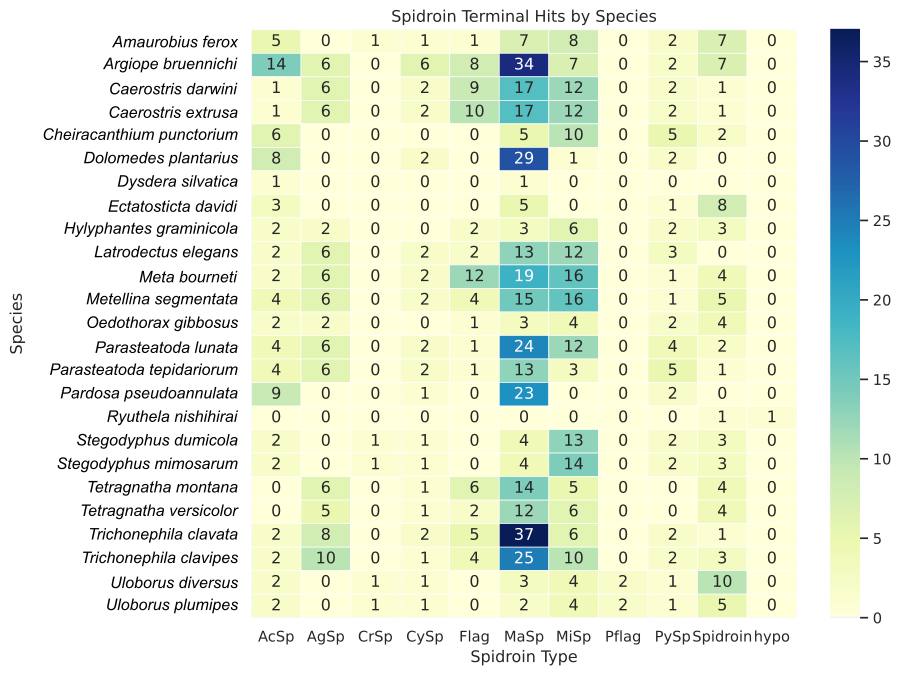


Figure S 21: Detected spidroin terminal regions. This figure does not discriminate between NTD and CTD. The terminal regions were joined so that a NTD and the next CTD form a full spidroin sequence. “hypo” stands for the hypothetical CTD found in *Ryuthela nishihirai*.


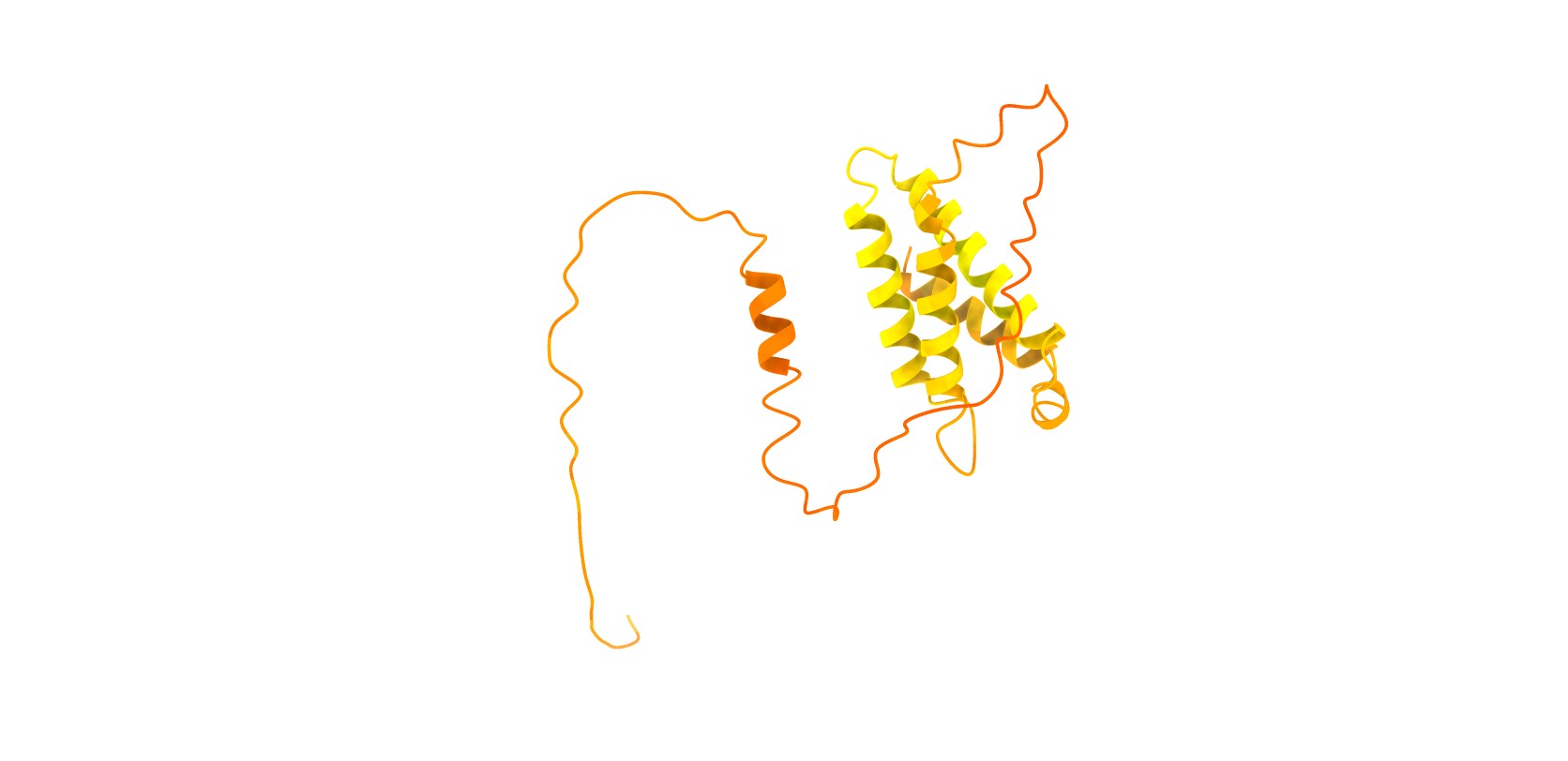


Figure S 22: Predicted structure of the presumed C-terminal region found in *Ryuthela nishihirai*. It looks similar to the known structure of other C-terminal regions e.g. Q2VLH4, Q2VLH3, A0A140DL57 in the AlphaFold Protein Structure Database and Jumper et al. (2021). Therefore, we think it is highly likely that it has es the same function as already known C-terminal regions.


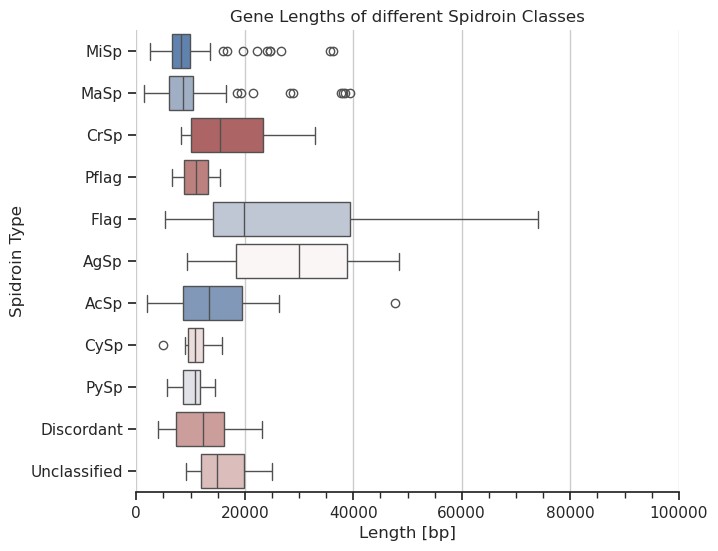


Figure S 23:Length distribution of all annotated spidroins in their respective spidroin classes. Abbreviations: AcSp - Aciniform, AgSp - Aggregate, CrSp - Cribellar, CySp - Cyllindrical, Flag - Flagelliform, MaSp - Major Ampullate, MiSp - Minor Ampullate, Pflag - Pseudoflagelliform, PySp - Pyriform, Spidroin - unclassified spidroin, disc. – Discordant (i.e. NTD and CTD were assigned to different classes).

References

Araujo, D., Schneider, M.C., Paula-Neto, E., Cella, D.M., 2024. The spider cytogenetic database. [www.arthropodacytogenetics.bio.br/spiderdatabase](http://www.arthropodacytogenetics.bio.br/spiderdatabase).

Datta, S.N., Chatterjee, K., 1983. Chromosome number and sex-determining system in fifty-two species of spiders from North-East India. Chromosome Information Service 35, 6–8.

Gurevich A, Saveliev V, Vyahhi N, Tesler G (2013) QUAST: quality assessment tool for genome assemblies. Bioinformatics, 29, 1072–1075.

Jumper, J., Evans, R., Pritzel, A., Green, T., Figurnov, M., Ronneberger, O., Tunyasuvunakool, K., Bates, R., Žídek, A., Potapenko, A., Bridgland, A., Meyer, C., Kohl, S.A.A., Ballard, A.J., Cowie, A., Romera-Paredes, B., Nikolov, S., Jain, R., Adler, J., Back, T., Petersen, S., Reiman, D., Clancy, E., Zielinski, M., Steinegger, M., Pacholska, M., Berghammer, T., Bodenstein, S., Silver, D., Vinyals, O., Senior, A.W., Kavukcuoglu, K., Kohli, P., Hassabis, D., 2021. Highly accurate protein structure prediction with AlphaFold. Nature 596 (7873), 583–589. 10.1038/s41586-021-03819-2.

Kumbıçak, Z., Ergene, S., Kumbıçak, Ü., Ekiz, E., 2014. A chromosomal analysis of five spider species (Araneae: Gnaphosidae, Miturgidae and Philodromidae) from Turkey. Caryologia 67 (2), 155–159. 10.1080/00087114.2014.931637.

Mittal, O.P., 1970. Karyological studies on the Indian spiders IX. Chromosome constitution in two cribellate species. Genetica 41 (1), 575–580. 10.1007/BF00958936.

Sharma, N., Parida, B.B., 1987. Study of chromosomes in spiders from Orissa. Pranikee 8 (197), 71.

Seppey M, Manni M, Zdobnov EM (2019) BUSCO: Assessing Genome Assembly and Annotation Completeness. Methods in Molecular Biology, 1962, 227–245.

Srivastava, M.D., Shukla, S., 1986. Chromosome number and sex-determining mechanism in forty-seven species of Indian spiders. Chromosome Information Service 41, 23–26.

Suzuki, S., 1949. Cytological studies of some spiders. Zoological Magazine 58, 89–90.

Suzuki, S., 1954. Cytological studies in spiders. III. Studies on the chromosomes of fifty-seven species of spiders belonging to seventeen families, with general considerations on chromosomal evolution. J Sci Hiroshima Univ B 15, 23–136.

Tanikawa, A., 2013. Phylogeny and genetic variation in the spiders of the genus *Ryuthela* (Araneae: Liphistiidae). Acta Arachnol. 62 (1), 41–49. 10.2476/asjaa.62.41.

Xu, X., Liu, F., Ono, H., Chen, J., Kuntner, M., Li, D., 2017. Targeted sampling in Ryukyus facilitates species delimitation of the primitively segmented spider genus *Ryuthela* (Araneae: Mesothelae: Liphistiidae). Zoological Journal of the Linnean Society 181 (4), 867–909. 10.1093/zoolinnean/zlx024.
